# Effects of first-step constraints on multi-muscle synergies and early postural adjustments during step initiation

**DOI:** 10.1007/s00221-026-07412-9

**Published:** 2026-10-20

**Authors:** Mateus Santos-Dias, Paulo B. de Freitas, Sandra M. S. F. Freitas, Mark L. Latash

**Affiliations:** 1 Interdisciplinary Graduate Program in Health Sciences, Universidade Cruzeiro do Sul, Rua Galvão Bueno, 868, Bloco B, 12º Andar, Liberdade, São Paulo, SP 01506-000 Brazil; 2 Master and Doctoral Program in Physical Therapy, Universidade Cidade de São Paulo, São Paulo, SP Brazil; 3 Department of Kinesiology, The Pennsylvania State University, University Park, PA USA

**Keywords:** Balance, Variability, Muscle activation, Motor control, Stability, Motor abundance

## Abstract

Step initiation requires the coordinated preparation of posture and movement, yet how first step constraints influence multi-muscle synergies stabilizing the center of pressure (COP), anticipatory synergy adjustments (ASAs), and early postural adjustments (EPAs) remains poorly understood. This study examined the effects of stepping surface height and step length on these outcomes in healthy young adults. Sixteen participants initiated a step under five conditions: level, step-up, step-down, short-step, and long-step. Surface electromyography was recorded from 16 leg and trunk muscles bilaterally, and COP coordinates were obtained from a force platform. The uncontrolled manifold approach was used to quantify multi-muscle synergies and ASAs stabilizing COP in the anteroposterior (AP) and mediolateral (ML) directions during steady-steady and around the foot lift-off {−25:+25 ms}. Multi-muscle synergies stabilizing COP-AP were present across all conditions, and their index did not differ significantly across stepping conditions, supporting the view that steady-state synergic organization reflects the demands of upright stance rather than anticipation of the upcoming step. Synergies stabilizing COP-ML were absent in all conditions. ASAs were not detected in any condition or direction, suggesting that step initiation relies more on EPA-based rather than synergy-level anticipatory mechanisms. Stepping surface height significantly influenced EPA onset timing and COP displacement magnitude in both directions, with the step-down condition producing earlier EPA onset and smaller displacements than the step-up condition. Step length did not significantly affect EPAs. These findings reveal a dissociation between synergic muscle organization and COP trajectory control and highlight the asymmetric postural demands of descending versus ascending steps during step initiation.

## Introduction

The control of body position during upright standing requires the continuous coordination of multiple muscles acting across several joints. This motor redundancy, i.e., the availability of more motor elements than strictly necessary to perform a given task, has traditionally been viewed as a computational problem for the central nervous system (CNS) (Bernstein 1967; reviewed in Prilitsky and Zatsiorsky, 2002). An alternative perspective, the principle of motor abundance (Latash 2012), treats large numbers of elements not as a problem but as an important feature of the body design that enables the CNS to ensure both stability and flexibility of movement patterns. Under this framework, the CNS organizes motor elements into coordinative structures, termed motor synergies, that stabilize task-relevant performance variables while preserving the flexibility to adapt to changing environmental and internal conditions (Latash 2012; Latash et al. 2007; Scholz and Schöner 1999).

The Uncontrolled Manifold (UCM) approach offers a method to quantify motor synergies by decomposing inter-trial variability of motor elements into two components: variance within the UCM (*V*_*UCM*_), which does not affect the task-relevant performance variable, and variance orthogonal to the UCM (*V*_*ORT*_), which affects the performance variable (Scholz and Schöner 1999, 2014). A synergy index (*ΔV*), computed as the normalized difference between *V*_*UCM*_ and *V*_*ORT*_, has been used as an index of stability of the corresponding performance variable. Positive *ΔV* values indicate that the variable is stabilized, whereas *∆V* ≤ 0 suggest the absence of synergic control for that performance variable (Park et al. 2012a; Scholz and Schöner 2014).

During upright standing, synergic control operates across multiple levels of the motor hierarchy, including muscle activations, joint kinematics, and muscle torques, to stabilize the center of pressure (COP, the point of application of the vertical ground reaction force) coordinates (Freitas and Duarte 2012; Hsu et al. 2007; Krishnamoorthy et al. 2003b, 2004; Park et al. 2016; Scholz et al. 2012). Changes in the COP coordinate generate time-varying moment of force acting on the center of mass (COM). In other words, at the level of mechanics, COP shifts can be viewed as a tool to induce COM shifts (Winter et al. 1996). The inherent instability of the upright posture, arising from a small base of support, a high COM, and a multisegmented body structure, makes the synergic control of COP coordinate essential for safe and efficient postural control. In the present study, the COP was selected as the task variable for the synergy analysis because it provides a low-dimensional mechanical measure of the forces and moments generated by the coordinated action of the muscles. This choice is also consistent with previous synergy analyses of step initiation that used COP as the task variable (Wang et al. 2005, 2006a; Falaki et al. 2023). Changes in COP seen in preparation to a whole-body action, such as step initiation, or reaction to a predictable perturbation, such as during catching and releasing objects, are closely linked to the concepts of early postural adjustments (EPAs) and anticipatory postural adjustments (ASAs), respectively.

Performing voluntary movements from a standing posture introduces additional demands on postural control, as the mechanical coupling between body segments generates forces, torques and joint configuration changes that must be anticipated and managed by the CNS. Studies of load release and arm raising during standing have shown that the synergy index computed for the COP coordinate drops approximately 200 ms before movement onset relative to steady-state levels, reflecting a transient reduction in the stability of this variable (de Freitas et al. 2022; Falaki et al. 2017; Freitas et al. 2020; Klous et al. 2011). These anticipatory synergy adjustments (ASAs) have been interpreted as a feedforward mechanism that reduces stability of a performance variable in preparation for its intended change (Latash & Huang 2015). Evidence further suggests that ASAs and APAs may represent partially independent components of movement preparation. Piscitelli et al. (2017) examined self-triggered unloading perturbations in which the timing of the perturbation was always known in advance, but its direction was either known or unknown to the participant. They showed that APAs were delayed and characterized by greater antagonist co-contraction when perturbation direction was unknown rather than known, whereas ASAs remained unchanged across these conditions, suggesting that APAs are more strongly associated with the specific mechanical requirements of the upcoming action, while ASAs may reflect a more general process relatively insensitive to such characteristics of a planned action as its direction (Piscitelli et al. 2017). This dissociation is particularly relevant in the context of step initiation, where the mechanical demands are more complex than in upper-limb tasks (e.g., arm lifting and load releas*e*). The transition from double- to single-limb support requires the CNS to simultaneously manage whole-body equilibrium and generate the propulsive forces needed to initiate a step, a context in which the relative contributions of APAs and ASAs to movement preparation remain to be fully characterized.

Step initiation from a standing posture represents a particularly informative model for studying the interplay between postural stability and voluntary movement. Prior to foot lift-off, the COP follows a characteristic trajectory: it first shifts toward the stepping foot, then reverses toward the stance foot while simultaneously moving backward (Brenière and Do 1991; Brenière et al. 1987; Crenna and Frigo 1991; Halliday et al. 1998). These EPAs starting 0.5–1 s prior to step initiation serve to unload the stepping limb and generate the forward moment of force necessary to initiate a step (Massion 1984). The present study focuses specifically on EPAs rather than APAs, a distinction that rests on the differential timing and function of these two components of postural preparation. APAs, as originally described, involve changes in postural muscle activation approximately 50–150 ms prior to a discrete, self-initiated or expected perturbation, and aim to generate forces and moments that counteract its anticipated destabilizing effects (Belenkiy et al. 1967; Bouisset and Zattara 1987; Krishnan et al. 2011, 2012). EPAs, in contrast, occur substantially earlier (approximately 500–1000 ms in advance) and reconfigure the body’s initial postural state in preparation for the upcoming voluntary action, rather than counteracting a specific perturbation (Krishnan et al. 2011, 2012; Massion 1984). Because step initiation in the present study was self-paced and voluntary, the anticipatory COP shifts are better characterized as EPAs, consistent with their role in preparing the body for the mechanical demands of stepping rather than counteracting an expected destabilizing event.

Falaki and colleagues examined multi-muscle synergies stabilizing the COP in the anteroposterior (AP) and mediolateral (ML) directions during step initiation in individuals with Parkinson’s disease (PD), older adults, and younger adults (Falaki et al. 2023). Their findings revealed that individuals with PD exhibited weaker steady-state synergies and smaller ASAs at foot lift-off compared with age-matched controls, and that older adults showed similar but less pronounced deficits relative to younger adults. Together, these findings highlight the sensitivity of synergic control mechanisms to the neural and biomechanical constraints of the stepping task, yet most of this evidence comes from level-surface conditions.

While step initiation has been studied extensively under level-surface conditions (Brenière and Do 1991; Brenière et al. 1987; Crenna and Frigo 1991; Halliday et al. 1998; Falaki et al. 2023), real-world locomotion frequently requires stepping onto surfaces at different heights or covering distances that deviate from a preferred stride length. Stepping upward or downward displaces the COM both forward and vertically, requiring greater angular excursions and joint moments in the lower limbs and pelvis compared with level stepping (McFadyen and Winter 1988; Nadeau et al. 2003). Studies have shown that the duration and amplitude of EPAs are reduced when stepping upward compared with level stepping, likely to facilitate the upward displacement of the COM (Gélat and Brenière 2000; Gélat and Le Pellec 2007). Similarly, step length constrains the magnitude of the required weight transfer and base-of-support displacement, and steps that deviate substantially from a preferred step length, whether shorter or longer, may impose additional mechanical and neuromuscular demands on the postural control system. Despite these observations, it remains unknown whether and how stepping surface height and step length influence the strength of multi-muscle synergies stabilizing the COP, the magnitude of ASAs, and the spatiotemporal characteristics of EPAs during step initiation. Addressing this gap is theoretically relevant because it can clarify whether synergic control of the COP and the spatiotemporal features of EPAs are governed by a common mechanism or can be dissociated when task mechanical demands change, informing current models of the neural organization of anticipatory postural control. This question also has practical relevance, as stepping onto surfaces of different heights or over different distances is common in daily locomotion and may be associated with increased fall risk in populations with compromised postural control, such as older adults and individuals with Parkinson’s disease (Falaki et al. 2023).

The present study addresses this gap by examining the effects of five stepping conditions (level, upward, downward, short, and long) on multi-muscle synergies, ASAs, and EPAs during step initiation in healthy young adults. We hypothesized that multi-muscle synergies stabilizing the COP in both the anteroposterior and mediolateral directions would be present during the steady-state phase (Hypothesis 1a) but would not differ across stepping conditions, reflecting the dependence of synergy strength on the demands of upright stance rather than on anticipation of the upcoming step (Hypothesis 1b). Extending the APA–ASA dissociation described above (Piscitelli et al. 2017) to step initiation across surfaces of different heights and distances, we hypothesized that ASAs would be present (Hypothesis 2a) but would show limited modulation in magnitude (i.e., the extent of the anticipatory decline in the synergy index, *ΔV*) across stepping conditions (Hypothesis 2b), whereas EPA characteristics would be sensitive to stepping constraints. Specifically, upward, downward, and long-step conditions were expected to produce larger preparatory adjustments than level and short-step conditions, given their greater mechanical and postural demands (Hypothesis #3).

## Methods

### Participants

Sixteen healthy young adults (8 women and 8 men; age: 27 ± 4 years; body mass: 70 ± 17 kg; height: 169 ± 11 cm) participated in this study. This sample size was not based on an a priori power calculation but was consistent with sample sizes used in previous studies from our laboratory employing similar experimental designs and outcome measures (e.g., Wang et al. 2005, 2006a; Falaki et al. 2023) and others using a single-step task (Gélat and Le Pellec 2007; Santos-Dias et al. 2026; Yamada and Shinya 2021).

Handedness and footedness were determined through self-report, using three questions, rather than a standardized inventory. Participants were asked which hand they used for writing and which foot they would use to kick a ball, as well as which foot they would prefer to use to initiate a forward step. All participants reported greater comfort initiating the step with the right foot. Inclusion required right-side preference on all three responses and no self-reported history of neurological or musculoskeletal conditions that could affect balance or gait. All participants reported normal or corrected-to-normal vision. They were recruited from the undergraduate and graduate student population at The Pennsylvania State University via email distributed through a laboratory mailing list and through announcements made during a university course and were financially compensated for their participation. Participants provided written informed consent prior to participation. The study was approved by the Institutional Review Board of The Pennsylvania State University.

### Apparatus

**Fig. 1:**
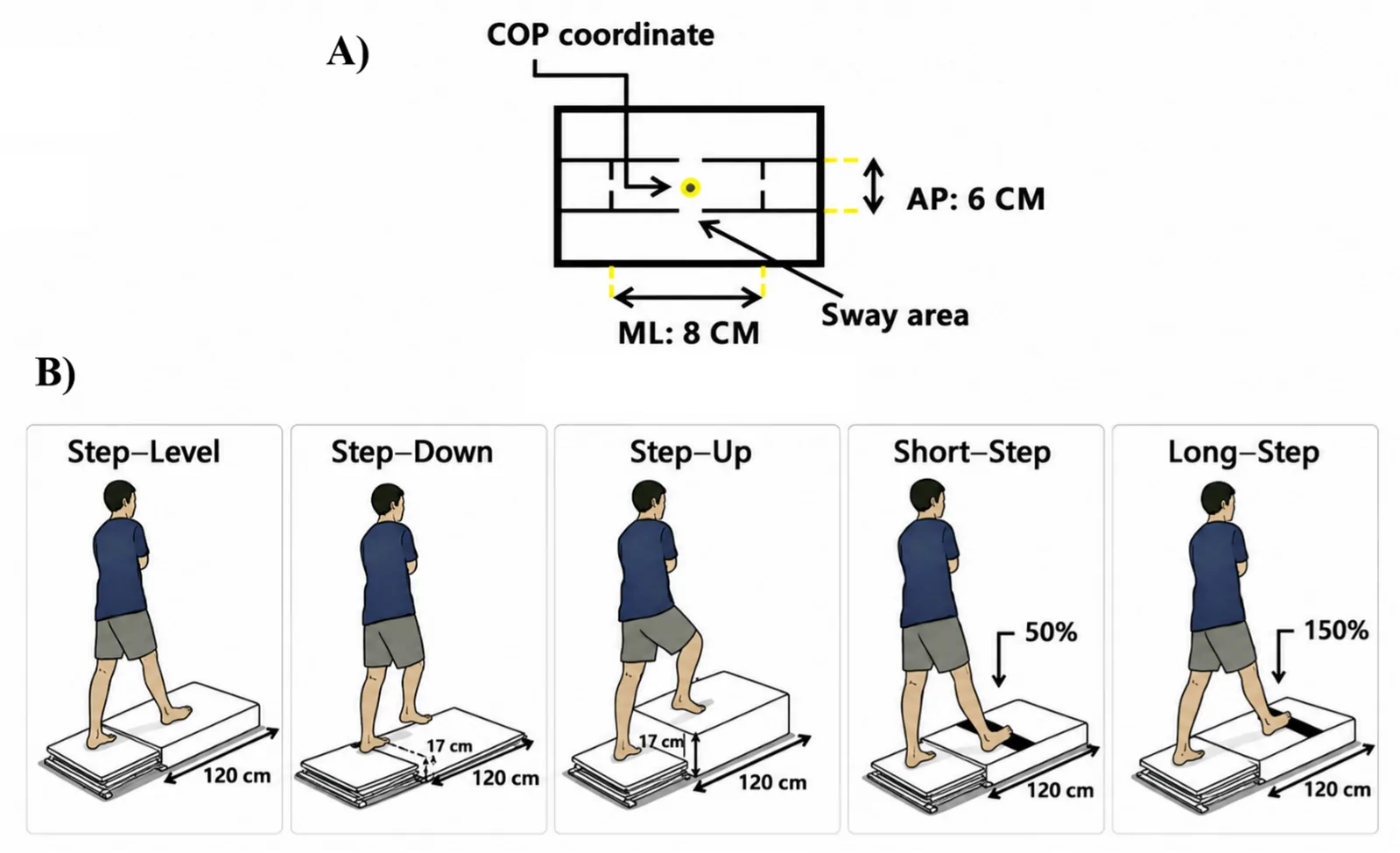
**A** Schematic illustration of the monitor display used in the present study, showing the screen dimensions (19 inches), the COP coordinate cursor, and the target displacement areas for voluntary sway in the anteroposterior (AP; 6 cm peak-to-peak) and mediolateral (ML; 8 cm peak-to-peak) directions. **B** Schematic representation of the five experimental stepping conditions: step-level, step-down, step-up, short-step, and long-step

Ground reaction forces and moments were recorded using a single force plate (OPTIMA, AMTI, Watertown, MA). COP coordinates in the anteroposterior (COP-AP) and mediolateral (COP-ML) directions were computed from the force plate data and displayed to participants as a 1-cm white circular cursor on a 19-inch computer monitor positioned 2 m in front of the participant at eye level (Fig. 1). Movements of COP-AP displaced the cursor vertically, whereas movements of COP-ML displaced it horizontally (Fig. 1B). Two wooden platforms (80 × 120 cm) were used, one with a height of 17 cm and another with a height of 34 cm to modify stepping height.

Surface electromyographic activity (EMG) was recorded from 16 muscles (8 bilateral pairs) using a 16-channel wireless system (Trigno Wireless EMG System, Delsys Inc., Natick, MA, USA): tibialis anterior (TA), soleus (SOL), biceps femoris (BF), rectus femoris (RF), vastus lateralis (VL), tensor fasciae latae (TFL), lumbar erector spinae (ESL), and rectus abdominis (RA). Electrode placement followed published guidelines (Criswell, 2010). Prior to placement, skin was shaved when necessary and cleaned with isopropyl alcohol to reduce impedance.

EMG and force-platform data were recorded continuously and synchronously at a sampling frequency of 1000 Hz using a custom-developed software program in LabVIEW (National Instruments., Austin, TX, USA). EMG signals were collected using a wireless electromyography system (Trigno Avanti, Delsys Inc.) configured with an embedded hardware band-pass filter of 20–450 Hz. Integration and stream control of EMG data within the LabVIEW environment were executed using the Delsys Trigno Development Kit (TDK). Signals from the force platform were digitized via an analog-to-digital conversion board (PCI-6225, 16-bit, National Instruments Co., Austin, TX, USA). Raw data were saved for offline processing.

### Experimental procedure

Testing was conducted in a laboratory environment, in a windowless room under constant standard fluorescent lighting. Before the main experimental conditions, participants completed two preliminary tasks: quiet standing (QS) and voluntary sway (VS), which were used, respectively, to obtain baseline EMG activity used later for correction and normalization of the EMG indices during the main trials and for the estimation of the muscle modes (M-modes) and Jacobian matrices required for the synergy analysis of the step-initiation task (see Data Processing). The entire experimental session lasted approximately 90 min. For all tasks, participants stood barefoot on the force platform with feet positioned shoulder-width apart and parallel to each other. Chalk outlines were drawn around the participants’ feet to ensure consistent foot placement across trials. The three tasks are described below in the order in which they were performed.

#### Quiet standing (QS) task

Participants stood as still as possible on the force platform for 65 s in a natural upright posture, with arms crossed over the chest and gaze directed toward the monitor. No visual feedback of COP coordinates was provided.

#### Voluntary sway (VS) task

In the VS task, participants produced continuous body sway movements around the ankle joint at 0.5 Hz, paced by an auditory metronome, for 35 s. These parameters were adopted from previous studies from our laboratory using the same M-mode/Jacobian estimation procedure (Freitas et al. 2020; Falaki et al. 2023; based on Danna-dos-Santos et al. 2007). A frequency of 0.5 Hz was chosen because it is slow enough to be performed as a controlled, voluntary sway rather than a fast, reactive or ballistic movement, while still eliciting sufficient modulation of muscle activity for the identification of muscle modes. The 35-s trial duration was chosen to yield a sufficient number of complete sway cycles for the M-mode and Jacobian estimation after excluding the initial and final segments of the trial (see below). Sway was performed separately in the anteroposterior (VS-AP) and mediolateral (VS-ML) directions, with two trials per direction completed in a block-randomized order. Visual feedback of COP-AP and COP-ML coordinates was provided throughout. Target sway amplitude was set at 6 cm peak-to-peak for AP movements and 8 cm peak-to-peak for ML movements, both centered around the origin (Fig. 1, top).

#### Step initiation task

Participants stood in a upright posture on the force platform, with their arms crossed in front of the chest, and leaned forward about the ankle joints to shift their COP-AP to a position located 3 cm anterior to the midpoint resting COP-AP coordinate. This target position was represented by a yellow marker and the COP-AP position was represented by a moving black circle, both continuously displayed on a monitor positioned at eye level. Participants were verbally instructed to keep the trunk and the hips in a neutral, non-flexed position, and to perform the forward lean using an ankle strategy only (i.e., rotation about the ankle joints, with minimal hip or trunk motion); consistency of the trunk orientation was visually monitored by the experimenter throughout each trial. This 3-cm anterior COP-AP displacement was actively maintained for approximately 2–3 s prior to the “go” command, rather than representing a continuous forward fall. It was designed to be small enough to preserve postural stability while still producing non-zero background muscle activity (cf. Piscitelli et al. 2017; Freitas et al. 2020; Falaki et al. 2023). The presence of background activity was verified qualitatively during data collection, based on a visible increase in the activation of posterior leg and trunk muscles relative to relaxed upright standing, without a quantitative amplitude threshold. Body lean was quantified by the anteroposterior COP displacement rather than by a segmental or joint angle change. Because forward lean angle was not directly measured, its average value cannot be reported. This is acknowledged as a limitation.

Participants were instructed to maintain the body leaning as steadily as possible for approximately 2–3 s while keeping both feet in full contact with the platform. After this period, the experimenter delivered a verbal command (“go”), and participants initiated the step. Participants were informed that the task was not intended to assess reaction and they were free to initiate stepping in a self-paced manner following the command.

Participants performed the stepping task under five conditions: step-level, step-up, step-down, short-step, and long-step (Fig. 1B). In all conditions, steps were initiated with the right (dominant) leg, followed by a step with the left (non-dominant) leg, after which participants immediately stopped. Each condition consisted of 24 trials. A 30-s and 2-min rest interval was provided between trials and conditions, respectively, to avoid muscle fatigue. The stepping task was organized into two blocks. In the first block, the step-level, step-up, and step-down conditions were performed in a counterbalanced order across participants. In the second block, the short-step and long-step conditions were performed in a counterbalanced order across participants; this block was always completed after the first block, because the individual step-length targets for the short-step and long-step conditions were derived from each participant’s mean step length in the step-level condition (see below).

In the level-step condition, participants stepped forward onto a wooden platform positioned at the same height as the force platform surface. In the step-down condition, participants stepped down from the force platform onto the floor; the force platform was mounted on a rigid metal support structure such that its surface was 17 cm above the floor. In the step-up condition, participants stepped forward onto a wooden platform positioned 17 cm above the force platform surface. This height falls within the typical range of residential stair riser heights (approximately 15–19 cm), which increases the ecological validity of the stepping task. Following these three conditions, participants completed the short-step and long-step conditions, both performed at the same surface height as the level-step condition.

Step length targets for the short-step and long-step conditions were individually determined based on each participant’s mean step length during the level-step condition. Step length was operationalized as the horizontal distance between the toe of the leading foot at its initial position and its landing position, measured with a tape placed along the stepping surface and recorded by the experimenter after each trial. The mean step length across the 24 level-step trials was calculated for each participant and used as the individual reference value.

In the short-step condition, participants were instructed to place the leading foot at a marker positioned at 50% of their individual reference step length (mean: 32.49 ± 6.5 cm from the initial toe position). In the long-step condition, the marker was positioned at 150% of the reference step length (mean: 96.0 ± 10.9 cm). In both conditions, participants were instructed to step forward and place the tip of the leading foot toe on the target marker on the wooden platform (Fig. 1B).

### Data processing

All data were processed offline using custom routines written in MATLAB (R2025b, MathWorks Inc., Natick, MA). Force plate data were digitally filtered using a fourth-order, zero-phase Butterworth low-pass filter with a cutoff frequency of 10 Hz, and COP-AP and COP-ML coordinates were subsequently computed from the filtered signals. EMG signals were band-pass filtered between 20 and 350 Hz using a fourth-order, zero-phase Butterworth filter, full-wave rectified, and smoothed with a 100-ms moving average window to obtain envelopes. These processing procedures were applied consistently across all three experimental tasks.

#### QS task

The QS task was used to obtain baseline EMG activity for all 16 muscles. To identify the period of greatest postural stability, the COP-AP signal was first detrended, and the root mean square (RMS) was computed using overlapping 10-s windows. The window with the lowest RMS value was selected as the most stable period. Mean EMG amplitude within the selected window was then computed for each channel and used as the baseline normalization reference for the step initiation task.

#### VS task

Data from the VS task provided two variables essential for processing the step initiation task: the peak EMG activation for each muscle (EMG-pk) and the muscle modes (M-modes), defined as groups of muscles exhibiting correlated activation patterns that act together as functional units (Krishnamoorthy et al. 2003a). M-modes were subsequently used to estimate the Jacobian matrix (**J**), which describes the linear relationship between changes in M-mode magnitudes (ΔM) and the resulting COP displacements (ΔCOP).

Sway cycles were defined separately for each direction. For AP sway, a cycle was defined as the time interval between two successive instances in which COP-AP crossed the origin while moving in the anterior direction. For ML sway, a cycle was defined as the interval between two successive instances in which COP-ML crossed the origin while moving to the right. To minimize the influence of sway initiation and termination, only cycles occurring during the middle 25 s of each trial were included in the analysis.

The filtered EMG signals were shifted 50 ms backward relative to the COP signal to account for electromechanical delay (Corcos et al. 1992). EMG and COP signals were then time-averaged using non-overlapping 50-ms windows.

For each participant, principal component analysis (PCA) with Varimax rotation was applied to the correlation matrix of the time-averaged EMG signals to identify patterns of coordinated muscle activation (Krishnamoorthy et al. 2003a; Danna-Dos-Santos et al., 2007). The number of components to retain was initially determined using the Kaiser criterion (Kaiser, 1960), retaining components with eigenvalues ≥ 1, and verified through visual inspection of the scree plots performed by a single evaluator. The two criteria were in agreement for all participants. Each retained component was required to have at least one muscle with a loading factor exceeding |0.5|. The retained components were addressed as M-modes. Across participants, four components consistently met these criteria. Additional components did not meet the loading criterion and did not provide a meaningful increase in the variance accounted for (VAF). Because the number of M-modes directly determines the dimensionality of the uncontrolled manifold (UCM) space and consequently affects the variance decomposition, four M-modes were retained for all participants to standardize the subsequent UCM analysis.

Separate Jacobian matrices were estimated for the AP and ML directions, using data from the AP and ML sway cycles, respectively. For each direction, ΔM (the change in magnitude of each of the four M-modes, computed within the same 50-ms windows used for EMG averaging) served as the predictor variables, and the corresponding ΔCOP (COP-AP or COP-ML) served as the dependent variable in a multiple linear regression performed across all sway cycles of that direction. The four resulting regression coefficients (one per M-mode) constituted the elements of the direction-specific Jacobian matrix (**J**-AP or **J**-ML). This matrix quantifies the linear relationship between changes in M-mode magnitude and the resulting COP displacement in that direction. Each direction-specific **J** matrix was subsequently applied to the step-initiation data to project M-mode variance onto the UCM and ORT subspaces (see below).

#### Step initiation task

Step initiation trials were time-aligned to the moment of right foot lift-off (t_0_), identified as the point at which COP-ML velocity exceeded 10% of its minimum value, corresponding to the end of the mediolateral COP shift toward the stance leg (McIlroy and Maki 1999; Melzer et al. 2007). The steady-state (SS) interval, referred to henceforth as the pre-step steady-state posture to distinguish it from the Quiet Standing (QS) task described above, was defined as the period from 1000 to 800 ms before t_0_, during which participants maintained their initial leaning posture with no visible anticipatory adjustments in EMG activity or COP coordinates. The absence of a marked increase in EMG activity during this interval was verified qualitatively through visual inspection of the EMG time series. COP stability during this interval was verified quantitatively: trials in which COP-AP deviated from the mean SS value by more than two standard deviations during this interval were excluded. Trials were additionally excluded if they contained EMG artifacts or faulty force platform recordings. The same set of retained trials was used for both the AP and ML analyses. Overall, 76.0% of all recorded stepping trials (1472 of 1937) were retained for analysis (24.0% excluded). Retention rates were similar across conditions: 74.5% for step-level, 77.8% for step-down, 78.2% for step-up, 75.9% for short-step, and 73.5% for long-step (mean number of usable trials per participant per condition: 18.4 ± 2.0, out of 24 trials).

For each trial, M-mode variations were demeaned (ΔM-demeaned = ΔM − ΔM-mean) and projected onto the uncontrolled manifold (UCM) and orthogonal (ORT) subspaces to compute variance per dimension (three dimensions for the UCM and one for the ORT) yielding *V*_*UCM*_ and *V*_*ORT*_, respectively. Mean values of *V*_*UCM*_ and *V*_*ORT*_ were calculated during the SS interval and within a 50-ms window centered at t_0_ (t_0_ − 25 ms to t_0_ + 25 ms) and were used to compute the synergy index within each interval. This 50-ms window was used, rather than a single time point at t_0_, to obtain a more stable estimate of variance by reducing the influence of instantaneous noise in the EMG and COP signals and for consistency with the window used in prior studies from our laboratory examining ASAs during step initiation (Falaki et al. 2023).

A synergy index was calculated to represent the relative difference between *V*_*UCM*_ and *V*_*ORT*_: *ΔV* = (*V*_*UCM*_ - *V*_*ORT*_)/V_TOT_, where V_TOT_ denotes total variance per dimension (DOF = 4). A value of *ΔV* greater than zero indicates the presence of a multi-muscle synergy stabilizing the COP coordinate through M-mode co-variation.

To normalize its distribution for statistical analysis, *ΔV* values were transformed using a modified Fisher z-transformation, yielding *ΔV*_Z_: *ΔV*_*Z*_ = 0.5 × ln [(*ΔV* + 4) / (1.33 − *ΔV*)] – 0.549, where 4 is the absolute value of the minimum possible ΔV, 1.33 is the maximum possible ΔV, and 0.549 is a constant corresponding to the value of *ΔV*_*Z*_ when ΔV = 0. Positive values of *ΔV*_*Z*_ (*ΔV*_*Z*_> 0) indicate the presence of multi-muscle synergies stabilizing COP coordinate.

The mean *ΔV*_*Z*_ during the SS interval was defined as *ΔV*_*Z*_ SS. The mean *ΔV*_*Z*_ around t_0_ was calculated and used as the synergy index at step initiation (*ΔV*_*Z*_ t_0_). The change in the synergy index leading up to step initiation was referred to as anticipatory synergy adjustment (ASA) and quantified as the difference between *ΔV*_*Z*_ SS and *ΔV*_*Z*_ t_0_ (*ΔΔV*_*Z*_ = *ΔV*_*Z*_ SS - *ΔV*_*Z*_ t_0_). All analyses were conducted separately for the anteroposterior and mediolateral directions using direction-specific M-mode sets and **J** matrices.

For each trial, the onset of early postural adjustments (*t*_*EPA*_) was identified from the COP-AP and COP-ML time series. COP-AP and COP-ML coordinates were normalized by subtracting their mean amplitudes calculated over a 200 ms baseline window prior to movement onset, setting the reference position to zero. For COP-AP, *t*_*EPA*_ was defined as the first instant at which the absolute COP-AP value exceeded 10% of the absolute COP-AP peak displacement. For COP-ML, *t*_*EPA*_ was identified by searching retrospectively from the instant of peak COP-ML displacement toward the swing foot and was defined as the last sample at which the COP-ML signal remained below 10% of the peak COP-ML amplitude (e.g., if peak displacement = 4 cm, threshold = 0.4 cm).

COP amplitude in the AP direction (*ΔCOP*_*AP*_) was computed as the displacement of COP-AP from its value at *t*_*EPA*_ to its value at t_0_: *ΔCOP*_*AP*_= COP_AP_(t_0_) − COP_AP_(t_EPA_). For the ML direction, two amplitude measures were calculated to characterize the biphasic nature of the mediolateral COP trajectory during step initiation. *ΔCOP*_*ML1*_ was defined as the displacement of COP-ML from t_EPA_ to its maximum value in the direction of the leading leg, reflecting the initial weight transfer toward the swing limb. *ΔCOP*_*ML2*_ was defined as the displacement of COP-ML from its maximum value in the direction of the leading leg to t_0_, reflecting the subsequent return toward the stance limb preceding foot lift-off.

### Statistical analysis

Statistical analyses were performed using JASP (version 0.98.0; JASP Team, Amsterdam, The Netherlands) and were organized into two blocks based on the experimental design. The first block examined the effect of stepping surface height on the outcome variables, comparing three conditions: Step-Up, Step-Level, and Step-Down. The second block examined the effect of step length, comparing Short-Step, Step-Level, and Long-Step conditions. In the second block, the Step-Level condition was relabeled Preferred Step Length (Pref-Step) to reflect its functional role as the length reference in that comparison. In both blocks, all outcome variables were analyzed separately for the AP and ML directions. The normality of all dependent variables was assessed using the Shapiro-Wilk test prior to parametric analyses.

To test for the presence of multi-muscle synergies during the steady-state phase (Hypothesis 1a) and for the existence of ASAs (Hypothesis 2a), one-sample t-tests were performed against zero on *ΔV*_*Z*_ SS and *ΔΔV*_*Z*_, respectively, separately for the AP and ML directions and for each experimental condition. To control for multiple comparisons, *p*-values were adjusted using the Benjamini-Hochberg false discovery rate (FDR) procedure applied separately within each family of related comparisons (m = 3, corresponding to the three experimental conditions in each block of comparisons). FDR-adjusted *p*-values are reported as p_FDR_ in the Results section. Cohen’s d was calculated as an estimate of effect size for all one-sample t-tests, with values of 0.2, 0.5, and 0.8 interpreted as small, medium, and large effects, respectively (Cohen 1988).

The effects of stepping condition on synergy strength (Hypothesis #1b) and ASA magnitude (Hypothesis #2b) were examined using one-way repeated-measures analyses of variance (RM-ANOVA) on *ΔV*_*Z*_ SS and *ΔΔV*_*Z*_, respectively, separately for the AP and ML directions. For the EPA-related variables (Hypothesis 3), one-way RM-ANOVAs were performed separately for the AP and ML directions on *t*_*EPA*_, *ΔCOP*_*AP*_, *ΔCOP*_*ML1*,_ *ΔCOP*_*ML2*_. For all RM-ANOVAs, Mauchly’s test was used to assess the assumption of sphericity; when sphericity was violated, the Greenhouse-Geisser correction was applied to the degrees of freedom. Where significant main effects were found, post hoc pairwise comparisons with Bonferroni correction were conducted to control for multiple comparisons. Effect sizes were reported as partial eta squared (η²p). The initial significance level was set at α = 0.05 for all statistical tests.

## Results

On average, 13.5 ± 3.5 sway cycles in the AP direction and 16.0 ± 3.6 cycles in the ML direction were used to perform PCA analysis to determine the M-modes. Four M-modes were identified for each participant, as described in the Methods section. M-modes in the AP direction accounted for 64.5 ± 3.7% of variance in the original EMG space, and M-modes in the ML direction accounted for 58.2 ± 5.1%. Separate **J** matrices were estimated for the AP and ML directions using multiple linear regression. For all participants, a full set of ΔM-AP jointly predicted ΔCOP-AP in a multiple regression model (adjusted R² = 0.77 ± 0.07) and the full set of ΔM-ML jointly predicted ΔCOP-ML (adjusted R² = 0.69 ± 0.01), confirming that the four M-modes identified for each direction accounted for a substantial proportion of the variance in the COP AP and ML coordinates.

Following, the results are presented in two subsections corresponding to the two experimental blocks: stepping surface height and step length variation. Within each section, UCM-based outcomes (synergy index and ASAs) are reported first, followed by EPA outcomes, with AP and ML directions presented in that order throughout.

### Stepping surface height

**Fig. 2:**
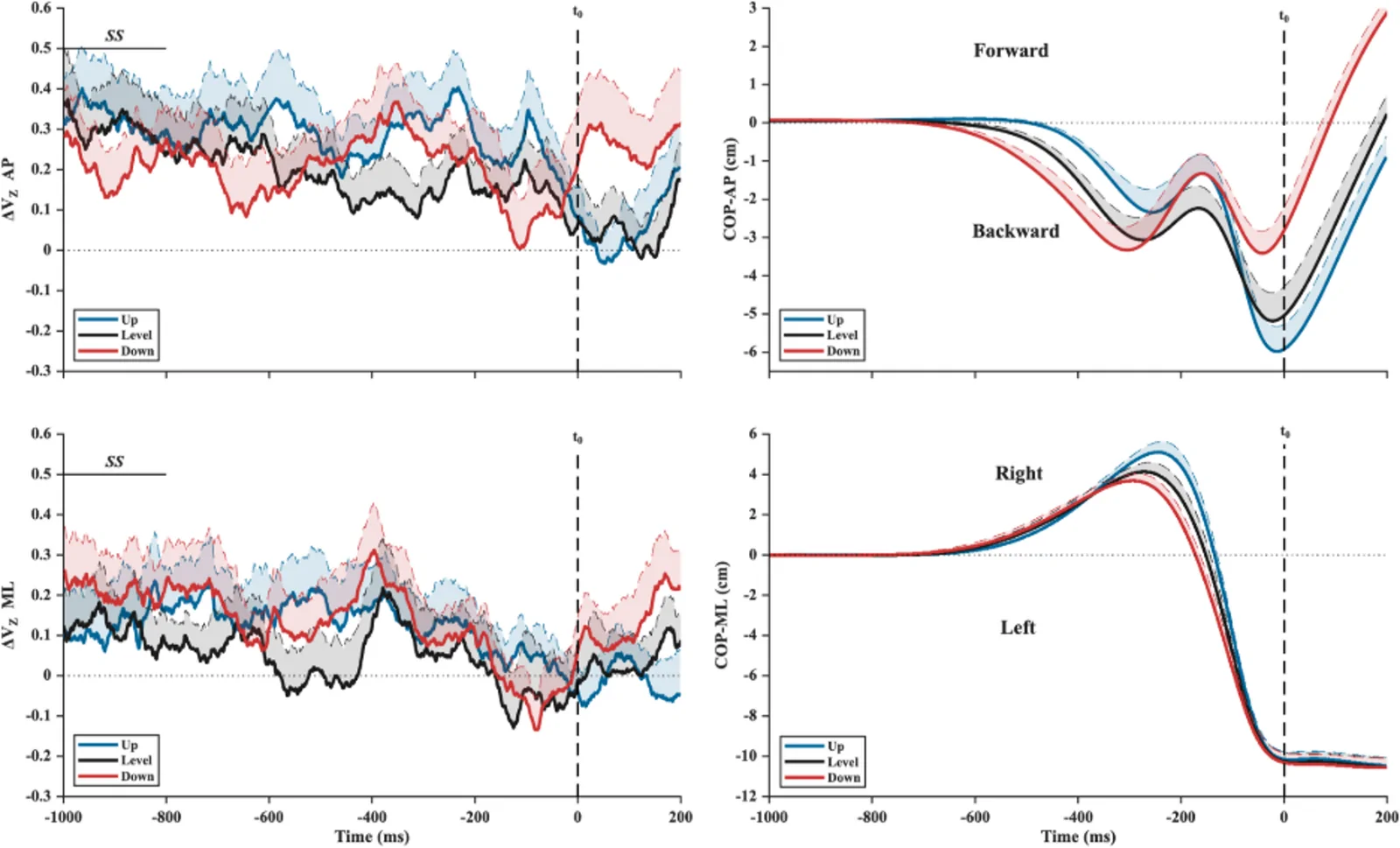
The mean time profiles of the synergy index (ΔV_Z_) and COP displacement during step initiation under the Step-up (blue), Step-level (black), and Step-down (red) conditions. Left panels show the time profiles of ΔV_Z_ in the anteroposterior (AP, top) and mediolateral (ML, bottom) directions. The right panels show the center of pressure (COP) displacement in the AP (top) and ML (bottom) directions. The vertical dashed line at time zero (t_0_) indicates foot lift-off. The horizontal solid line labeled “SS” indicates the steady-state interval. Shaded regions represent one standard error

**Fig. 3:**
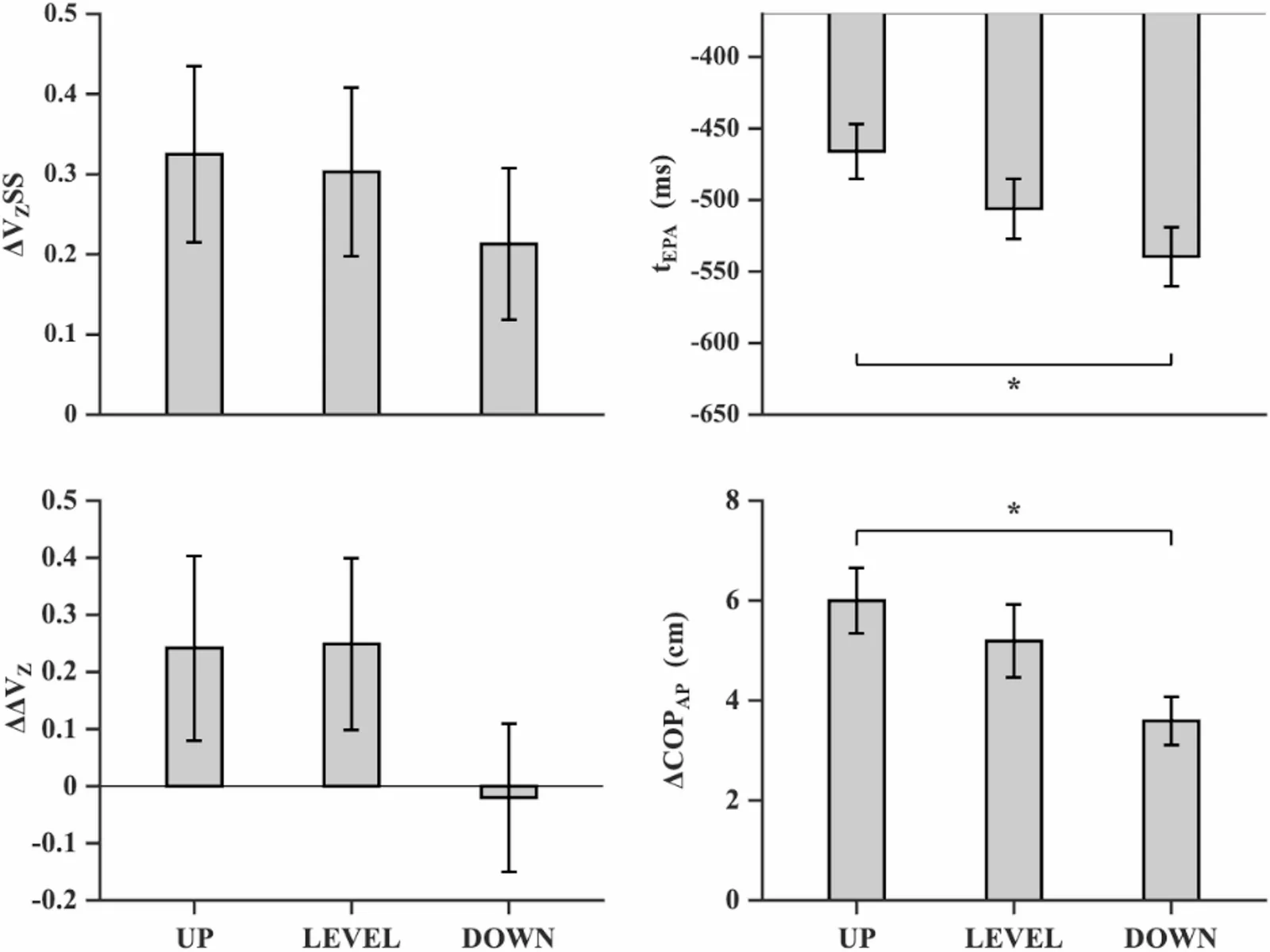
Mean and standard error of the mean for ΔV_Z_ SS (top, left), ΔΔV_Z_ (bottom, left), t_EPA_ (top, right), and ΔCOP_AMP_ (bottom, right) for each stepping surface height (up, level, and down) in anteroposterior direction

Figure 2 shows time-series of *ΔV*_*Z*_ (left panels) and COP displacement (right panels) for the three stepping surface height conditions in AP (top panels) and ML (bottom panels) directions. As illustrated in Figs. 2 and 3 (top, left), *ΔV*_*Z*_ SS in AP direction was greater than zero in all three conditions. One-sample *t*-tests confirmed the presence of multi-muscle synergies stabilizing COP-AP during the steady-state phase: Step-Level (t_15_ = 3.13, p_FDR_ = 0.014, d = 0.78), Step-Up (t_15_ = 2.99, p_FDR_ = 0.014, Cohen’s d = 0.75), and Step-Down (t_15_ = 2.15, p_FDR_ = 0.048, d = 0.54). The one-way RM-ANOVA revealed no significant main effect of stepping surface height on synergy strength (*ΔV*_*Z*_ SS: F_2, 30_ = 0.53, *p* = 0.592, η² = 0.03), indicating that synergy strength during the steady-state phase did not differ across conditions.

Regarding ASAs in the AP direction, one-sample t-tests indicated that *ΔΔV*_*Z*_ did not differ significantly from zero in any condition (Step-Level: t_15_ = 1.65, p_FDR_ = 0.165, d = 0.48; Step-Up: t_15_ = 1.86, p_FDR_ = 0.179, d = 0.41; and Step-Down: t_15_ = − 0.1, p_FDR_ = 0.926, d = − 0.03), indicating that ASAs were not detected in any conditions (Fig. 2, bottom, left). The one-way RM-ANOVA revealed no significant main effect of stepping surface height on ASA magnitude (*ΔΔV*_*Z*_: F_2, 30_ = 3.15, *p* = 0.057, η² = 0.17).

With respect to EPA, a one-way RM-ANOVA revealed a significant main effect of stepping surface height on EPA onset timing (*t*_*EPA*_: F_2, 30_ = 6.1, *p* = 0.006, η² = 0.31, Fig. 3, top, right). As illustrated in Fig. 2 (top, right) and confirmed by post-hoc pairwise comparisons with Bonferroni correction, *t*_*EPA*_ occurred earlier in the Step-Down condition than in the Step-Up condition (*p* = 0.008), with neither condition differing significantly from Step-Level.

A significant main effect of stepping surface height was also found for *ΔCOP*_*AP*_ (F_2, 30_ = 9, *p* < 0.001, η² = 0.38, Fig. 3, bottom, right). As shown in Fig. 2 (top, right) and supported by pairwise comparisons, *ΔCOP*_*AP*_ was lower in the Step-Down condition than in the Step-Up condition (*p* < 0.001), while the Step-Level condition did not differ significantly from either condition.

**Fig. 4:**
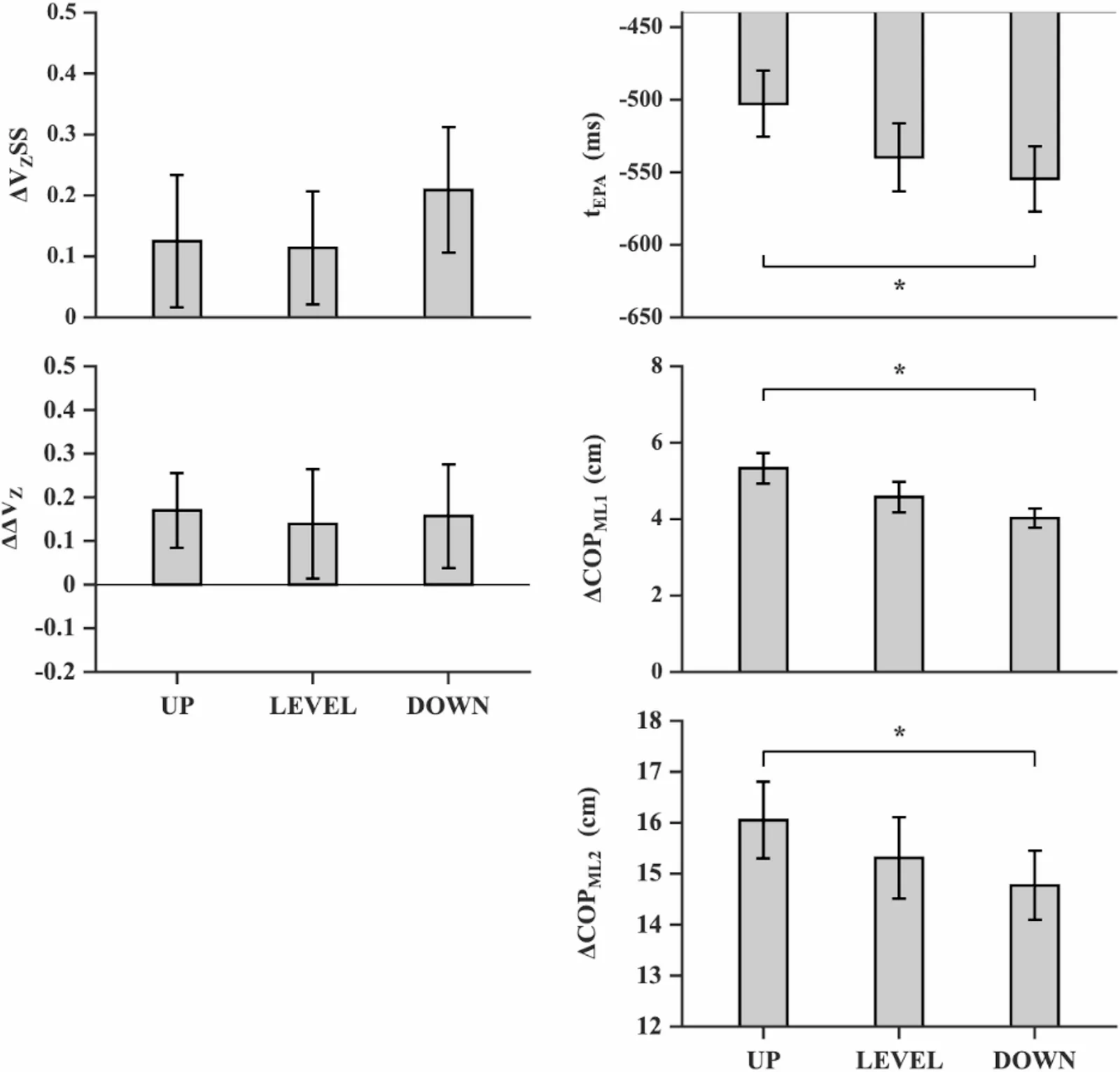
Mean and standard error of the mean for ΔV_Z_ SS (top, left) and ΔΔV_Z_ (bottom, left), t_EPA_ (top, right), ΔCOP_ML1_ (middle, right) and ΔCOP_ML2_ (bottom, right) for each stepping surface height condition in mediolateral direction

For the ML direction (Fig. 2, bottom panels and Fig. 4), one-sample t-tests did not confirm the presence of multi-muscle synergies stabilizing COP-ML during the steady-state phase in any condition: Step-Level (t_15_ = 1.24, p_FDR_ = 0.269, d = 0.31), Step-Up (t_15_ = 1.15, p_FDR_ = 0.269, d = 0.29), and Step-Down (t_15_ = 2.04, p_FDR_ = 0.18, d = 0.51). In addition, the one-way RM-ANOVA revealed no significant main effect of stepping surface height on synergy strength (*ΔV*_*Z*_ SS: F_2, 30_ = 0.61, *p* = 0.549, η² = 0.04), indicating that synergy strength during the steady-state phase did not differ across conditions.

Regarding ASAs in the ML direction (Fig. 4, bottom, left), one-sample t-tests indicated that ΔΔV_Z_ did not differ significantly from zero in any condition: Step-Level (t_15_ = 1.11, p_FDR_ = 0.284; d = 0.28); Step-Up (t_15_ = 2, p_FDR_ = 0.195, d = 0.5); and Step-Down (t_15_ = 1,32, p_FDR_ = 0.284, d = 0.33), suggesting that ASAs were not consistently present across conditions (Fig. 2, bottom, left). The one-way RM-ANOVA revealed no significant main effect of stepping surface height on ASA magnitude in ML direction (*ΔΔV*_*Z*_: F_2, 30_ = 0.03, *p* = 0.972, η² < 0.01); indicating that ASA magnitude did not differ across stepping surface height conditions.

Regarding the EPA in the ML direction (Fig. 4, right panels), the one-way RM-ANOVA revealed a significant main effect of stepping surface height on EPA onset timing (*t*_*EPA*_: F_2, 30_ = 4.2, *p* = 0.025, η² = 0.22). As illustrated in Fig. 2 (bottom, right) and 4 (top, right) and confirmed by post hoc pairwise comparisons, *t*_*EPA*_ in ML direction occurred earlier in the Step-Down condition than in the Step-Up condition (*p* = 0.005), with no significant difference between Step-Level and either condition.

A significant main effect of stepping surface height was also found for *ΔCOP*_*ML1*_ (F_2, 30_ = 9.65, *p* < 0.001, η² = 0.39). As shown in Fig. 2 (bottom, right) and 4 (middle, right) and supported by post hoc comparisons, *ΔCOP*_*ML1*_ was smaller in the Step-Down condition than in the Step-Up condition (*p* < 0.001), with no significant differences between Step-Level and either condition.

A significant main effect of stepping surface height was additionally found for *ΔCOP*_*ML2*_ (F_2, 30_ = 5.4, *p* = 0.01, η² = 0.26, Fig. 4, bottom, right). Pairwise comparisons showed that *ΔCOP*_*ML2*_ was lower in the Step-Down condition than in the Step-Up condition (*p* = 0.004), with no significant differences between Step-Level and either condition.

### Step length

**Fig. 5:**
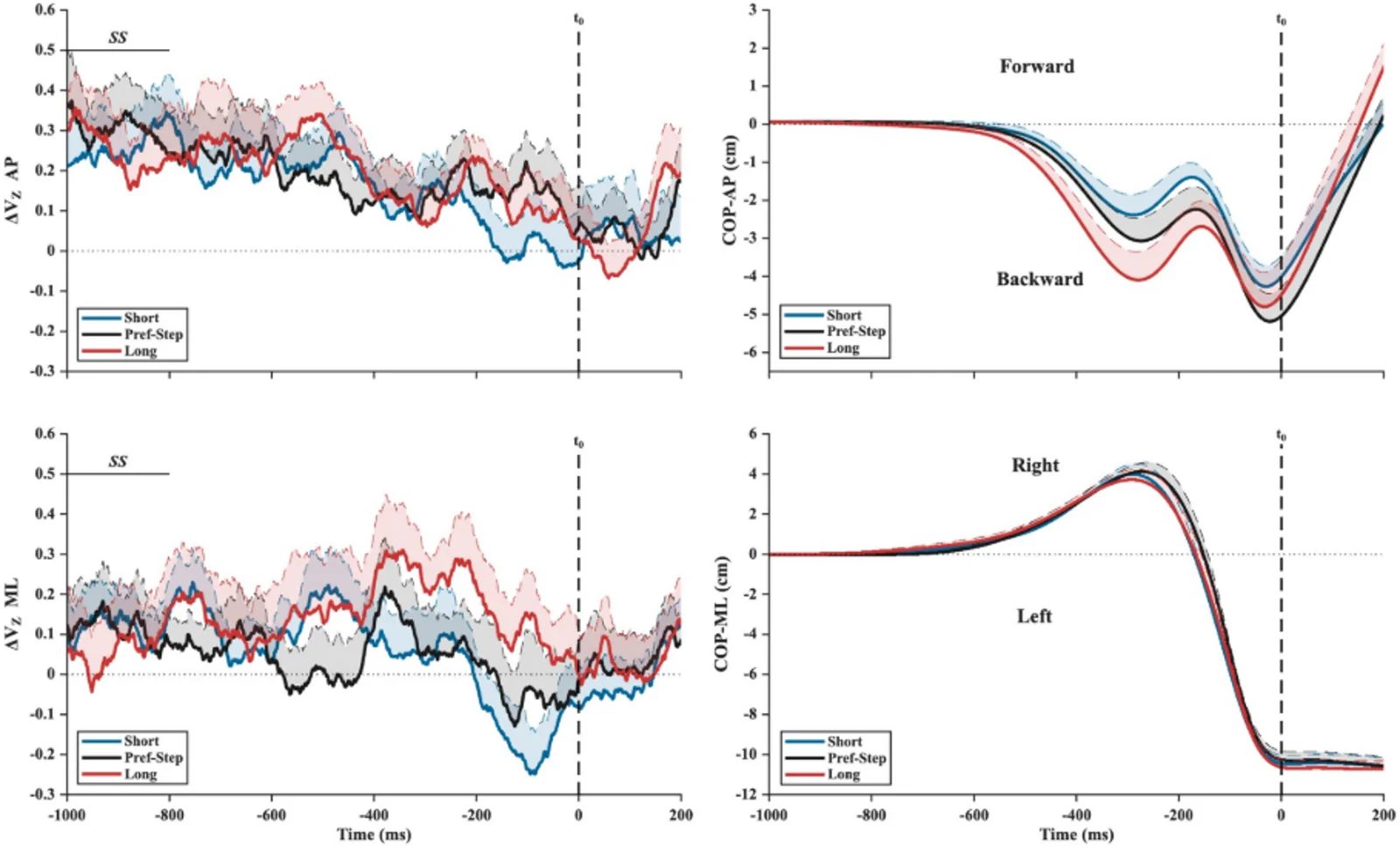
The mean time profiles of the synergy index (ΔV_Z_) and COP displacement during step initiation under the short-step (blue), preferred step length (Pref-Step, black), and long-step (red) conditions. Left panels show the time profiles of ΔV_Z_ in the anteroposterior (AP, top) and mediolateral (ML, bottom) directions. The right panels show the center of pressure (COP) displacement in the AP (top) and ML (bottom) directions. The vertical dashed line at time zero (t_0_) indicates foot lift-off. The horizontal solid line labeled “SS” indicates the steady-state interval. Shaded regions represent one standard error

**Fig. 6:**
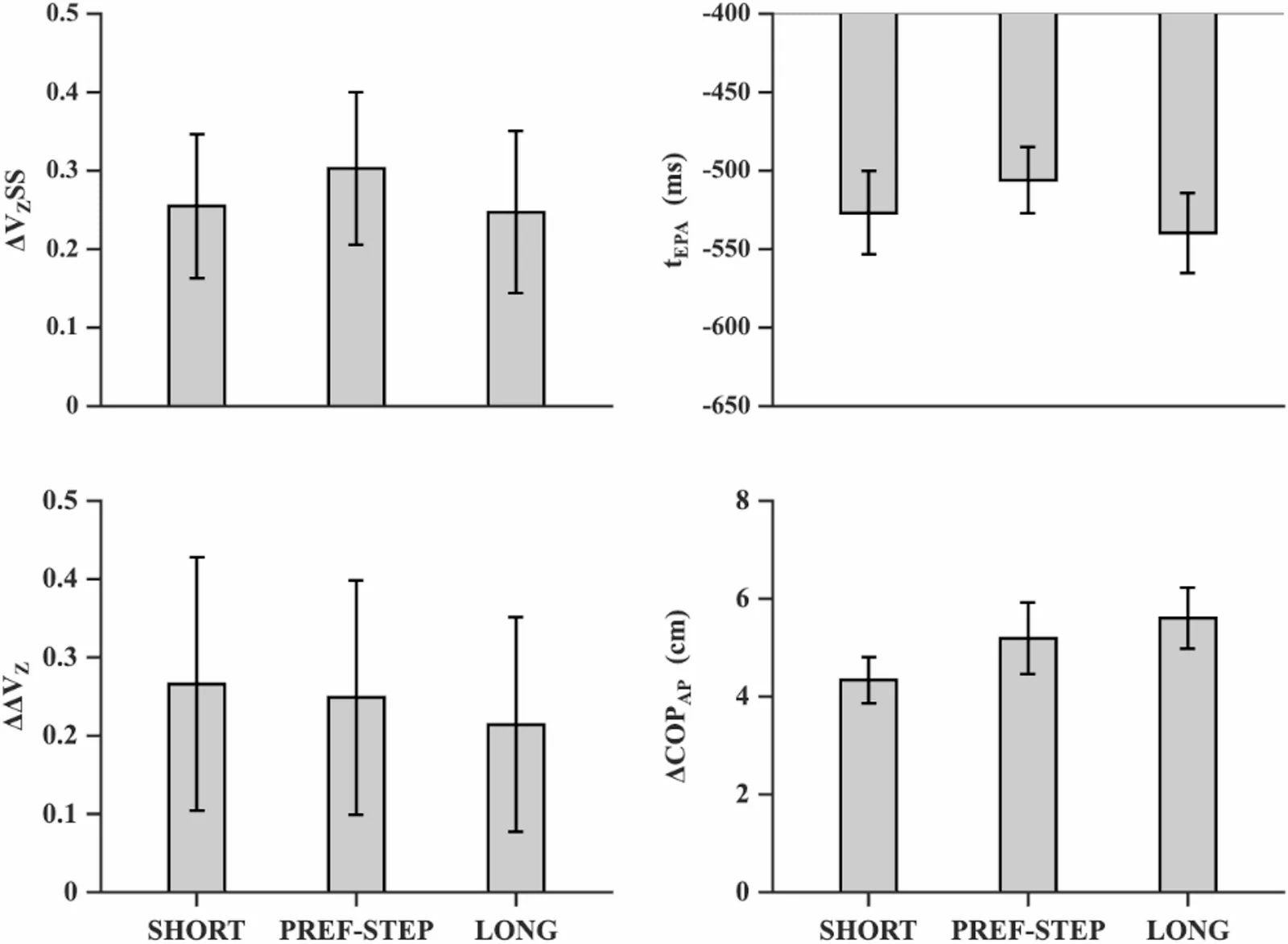
Mean and standard error of the mean for ΔV_Z_ SS (top, left), ΔΔV_Z_ (bottom, left), tEPA (top, right), and COP_AMP_ (bottom, right) for each step length condition (short step, preferred step length, and long step) in anteroposterior direction

Figure 5 shows time-series of ΔV_Z_ (left panels) and COP displacement (right panels) for the three stepping length conditions in AP (top panels) and ML (bottom panels) directions. As illustrated in Figs. 5 and 6 (top, left), *ΔV*_*Z*_ SS in AP direction was greater than zero in all three stepping length conditions. One-sample t-tests confirmed the presence of multi-muscle synergies stabilizing COP-AP during the steady-state phase: Pref-Step (t_15_ = 3.13, p_FDR_ = 0.021, d = 0.78); Short-Step (t_15_ = 2.76, p_FDR_ = 0.021, d = 0.69); and Long-Step (t_15_ = 2.39, p_FDR_ = 0.03, d = 0.6). The one-way RM-ANOVA revealed no significant main effect of step length on synergy strength (*ΔV*_*Z*_ SS: F_2, 30_ = 0.23, *p* = 0.794, η² = 0.02) indicating that synergy strength during the steady-state phase did not differ across conditions.

Regarding ASAs (Fig. 6, bottom, left), one-sample t-tests indicated that *ΔΔV*_*Z*_ did not differ significantly from zero in any condition (Pref-Step: t_15_ = 1.65, p_FDR_ = 0.138, d = 0.41; Short-Step: t_15_ = 1.64, p_FDR_ = 0.138, d = 0.41; Long-Step: t_15_ = 1.57, p_FDR_ = 0.138, d = 0.39), indicating that significant ASAs were not detected in the AP direction for all stepping length conditions. Also, one-way RM-ANOVA revealed no main effect of stepping length on ASA magnitude (*ΔΔV*_*Z*_: F_2, 30_ = 0.07, *p* = 0.937, η² < 0.01).

In terms of EPA, one-way RM-ANOVAs revealed no main effect of step length on EPA onset timing (*t*_*EPA*_: F_2, 30_ = 1.67, *p* = 0.204, η² = 0.1, Fig. 6, top, right), nor on *ΔCOP*_*AP*_ (F_2, 30_ = 2.8, *p* = 0.077, η² = 0.16, Fig. 6, bottom; right).

**Fig. 7:**
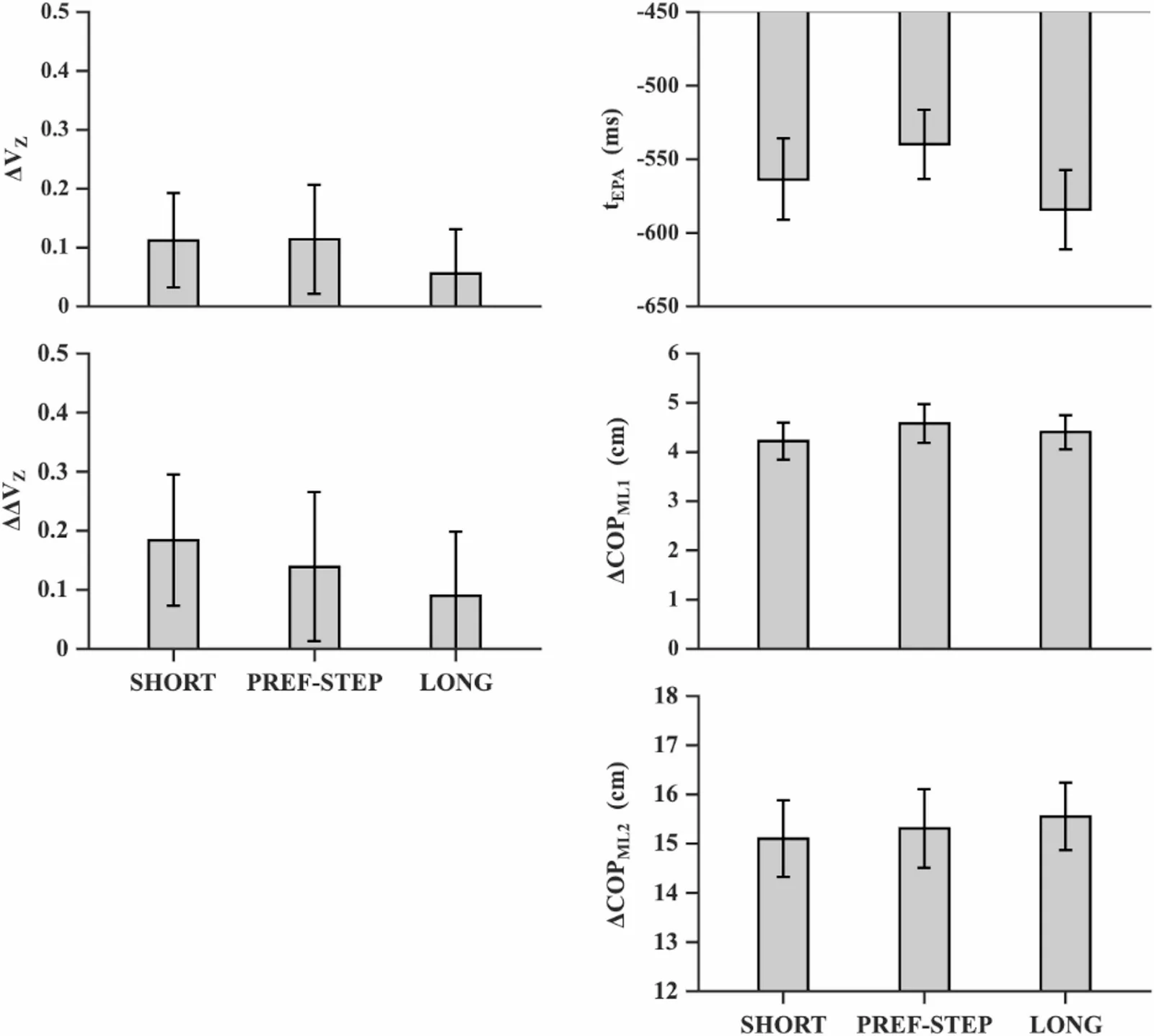
Mean and standard error of the mean of ΔV_Z_ SS (top, left), ΔΔV_Z_ (bottom, left), t_EPA_ (top, right), ΔCOP_ML1_ (middle, right) and ΔCOP_ML2_ (bottom, right) for each step length condition (short step, preferred step length, and long step) in mediolateral direction

For the ML direction (Fig. 7), one-sample t-tests did not confirm the presence of multi-muscle synergies stabilizing COP-ML during the steady-state phase in any condition: Pref-Step (t_15_ = 1.24, p_FDR_ = 0.351, d = 0.31); Short-Step (t_15_ = 1.4, p_FDR_ = 0.351, d = 0.35); and Long-Step (t_15_ = 0.74, p_FDR_ = 0.47, d = 0.18). Also, the one-way RM-ANOVA revealed no significant main effect of stepping length on synergy strength (*ΔV*_*Z*_ SS: F_2, 30_ = 0.34, *p* = 0.713, η² = 0.03), indicating that synergy strength during the steady-state phase did not differ across conditions.

Regarding ASAs in the ML direction (Fig. 7, bottom left), one-sample t-tests indicated that *ΔΔV*_*Z*_ did not differ significantly from zero in any condition: Pref-Step (t_15_ = 1.11, p_FDR_ = 0.417, d = 0.28); Short-Step (t_15_ = 1.67, p_FDR_ = 0.348, d = 0.42); and Long-Step (t_15_ = 0.83, p_FDR_ = 0.417, d = 0.21), suggesting that ASAs were not consistently present across conditions. Also, one-way RM-ANOVA revealed no main effect of stepping length on ASA magnitude (*ΔΔV*_*Z*_: F_2, 30_ = 0.284, *p* = 0.755, η² < 0.01).

For EPA in the ML direction, one-way RM-ANOVA showed no significant main effect of step length on the EPA onset timing (*t*_*EPA*_: F_2, 30_ = 3.22, *p* = 0.054, η² = 0.18). Furthermore, no main effect of step length was found for *ΔCOP*_*ML1*_ (F_2, 30_ = 1.06, *p* = 0.36, η² = 0.07) and *ΔCOP*_*ML2*_ (F_2, 30_ = 1.33, *p* = 0.278, η² = 0.08).

## Discussion

This study explored the influence of first-step constraints on multi-muscle synergies stabilizing the center of pressure, anticipatory synergy adjustments (ASAs), and early postural adjustments (EPAs) during step initiation. Specifically, we investigated whether stepping surface height and step length affected synergy strength during the steady-state phase preceding movement onset, the magnitude of ASAs, and the timing and magnitude of EPA-related COP displacements. The results partially supported our hypotheses. Consistent with Hypothesis 1a, multi-muscle synergies stabilizing COP-AP were present during the steady-state phase across all stepping surface height and step length conditions, supporting that the neuromuscular system organizes muscle activation into functional units to stabilize the anteroposterior COP regardless of the upcoming task demands. However, synergies stabilizing COP-ML were absent across all conditions and experimental blocks. Thus, although the presence of COP-AP synergies was consistent with our hypothesis, the absence of COP-ML synergies indicates that the predicted organization was not observed across both COP directions. This finding will be addressed in a dedicated section below. Consistent with Hypothesis 1b, synergy strength did not differ significantly across conditions in either experimental block in both directions, supporting the notion that steady-state postural organization reflects the demands of upright stance rather than anticipation of the upcoming step. Contrary to Hypothesis 2, ASAs were not significant in any condition or direction, suggesting that the neuromuscular system did not modulate multi-muscle synergies in anticipation of the upcoming step regardless of its mechanical demands. Hypothesis 3 received partial support: stepping surface height influenced both EPA onset timing and COP displacement magnitude in the AP and ML directions, with the Step-Down condition producing earlier onset but lower displacements than Step-Up, while Step-Level did not differ significantly from either condition. Step length, in contrast, did not significantly affect EPA onset or COP displacement.

### Two aspects of feed-forward control in step preparation

When the central nervous system (CNS) issues neural commands for an action, its results cannot be predicted perfectly because of the spontaneous changes in the body state and poorly predictable external forces. Nevertheless, prediction based on experience is a major component of everyday actions reflected in feed-forward adjustments that optimize the body state for the planned action (early postural adjustments, EPAs, Krishnan et al. 2011) and minimize the effects of predicted possible perturbations on postural task components (anticipatory postural adjustments, APAs, reviewed in Massion 1992). EPAs can be observed as early as 0.5–1 s prior to the action initiation, while APAs are more tightly linked to the action and are seen about 50-150 ms prior to the action initiation. While processes of preparation to a step have been commonly referred to as APAs, it seems more appropriate to address them as EPAs, similar to postural adjustments to other planned whole-body movements such as quick whole-body sway (Wang et al. 2006b).

While both EPAs and APAs can be seen in averaged over trials time profiles of EMG and mechanical variables (e.g., COP), another class of feed-forward adjustments is associated with changes not in the magnitudes of EMG and mechanical variables but in their stability. These are referred to as anticipatory synergy adjustments (ASAs), which have been quantified during whole-body tasks including keeping vertical posture and step initiation (Klous et al. 2010; Krishnan et al. 2011; Falaki et al. 2023). ASAs in such tasks can be detected 200-300 ms prior to action initiation. They attenuate the stability of a salient performance variable in anticipation of its quick change and thus avoid problems associated with a self-initiated quick change of a strongly stabilized variable. In other words, ASAs allow the neural controller to avoid fighting its own synergies.

Piscitelli and collaborators (2017) shown that ASAs do not show significant changes with features of the upcoming postural perturbation, such as predictability of its direction, whereas APAs show major changes, which include both timing and magnitude characteristics (Piscitelli et al. 2017). These authors concluded that the CNS had two types of feedforward control tools that are used to prepare for a predictable perturbation, a more universal one (ASAs), which is effective independently of the direction and magnitude of shift of the salient performance variable, and more task-specific ones (APAs), which show adjustments to the planned action mechanics.

Our study provides evidence for a similar contrast between ASAs and EPAs in tasks that require a self-initiated whole-body action. Indeed, changes in step conditions were reflected in significant changes in EPA characteristics, while there were no comparable significant changes in the synergy index reflecting COP stability. These results corroborate the distinction between feed-forward adjustments that change salient variables and those that modify their stability.

### The neural control of posture and its adjustments

**Fig. 8:**
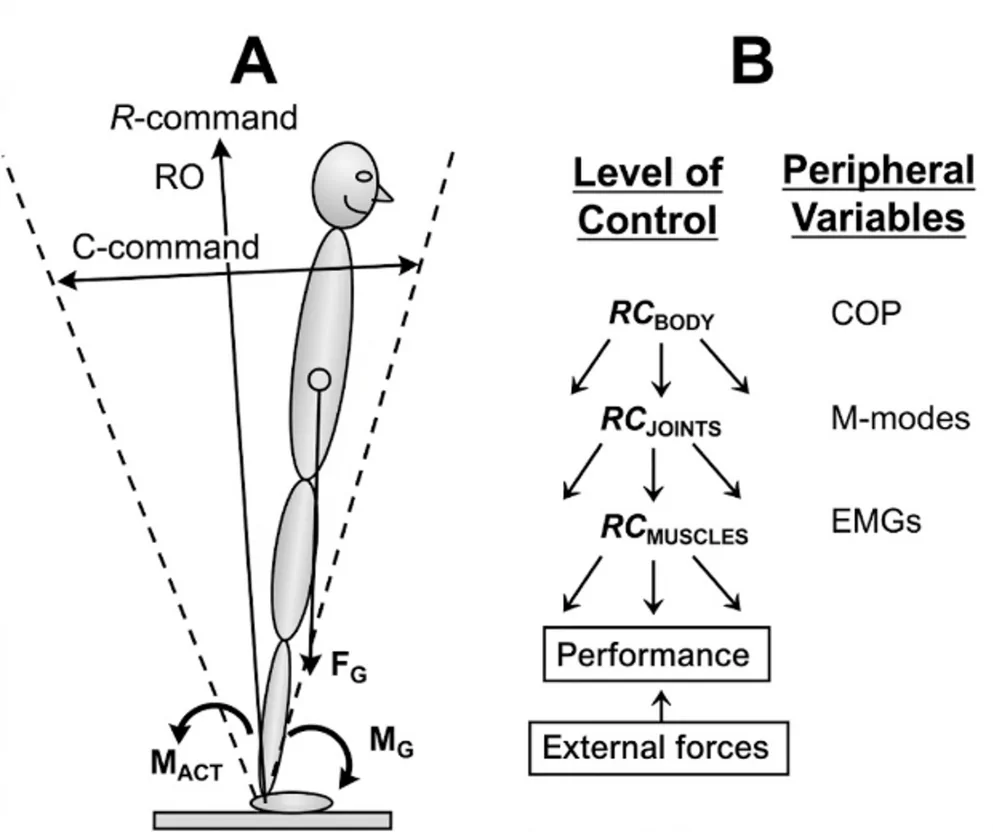
**A** The scheme of control of the body during quiet standing. The center of mass typically projects in from of the ankle joints, and the gravity force (F_G_) produced a moment (M_G_). It is counteracted by active moment (M_ACT_), which is a consequence of the difference between the actual body orientation and referent orientation (RO) defined by the R-command. The C-command translates this spatial variable into moment units. **B** Three levels of hypothetical control are shown related to the whole body, joint configuration, and muscle activation. The {R; C} commands are reflected in corresponding peripheral variables shown on the right.

Although the control of posture and movement is commonly described and studied in terms of peripheral variables, such as forces, joint kinematics, and muscle activations, these variables cannot be prescribed by the brain, given the unpredictability of external forces, spontaneous fluctuations in the CNS state, and the action of reflex loops from peripheral receptors (Bernstein 1947; Feldman 2015). One of the approaches to motor control implies that the CNS operates with spatial referent coordinates for the effectors, which can be adequately described as combinations of two basic commands, reciprocal (R-command) and coactivation (C-command), an {R; C} pair specified for each kinematic degree of freedom, linear or rotational (Latash 2010; Latash 2016; Feldman 2015). The concept of {R; C} commands was originally developed to describe the neural control of a single joint spanned by a pair of agonist–antagonist muscles (Feldman 1986). Later, the concept was generalized for any effector up to the whole body (Latash 2010). For any kinematic degree of freedom, muscles can be grouped by their combined action on a selected performance variable into those that contribute to its motion in a particular direction and in the opposite direction, agonists and antagonists. Such {R; C} pairs can be introduced, in particular for motion of the body in the anteroposterior and mediolateral directions. At the level of mechanics, the R-command defines referent coordinate for the effector, i.e., a coordinate that the effector would achieve in the absence of external forces. The C-command defines apparent stiffness of the effector (cf. Latash and Zatsiorsky 1993), i.e., its resistance to changes in the external forces. This is illustrated schematically in Fig. 8A for the anteroposterior direction. Note that, to keep balance, the moment of force generated by the muscles (M_ACT_) has to balance the moment generated by the gravity force (M_G_). To produce a required magnitude of M_ACT_, the brain can use various {R; C} pairs. For example, a small R-command (small deviation of the referent orientation, RO, from the actual body orientation, AO) can produce a large M_ACT_ if it is accompanied by a large C-command leading to a large apparent rotational stiffness, k. Indeed, in a linear approximation M_ACT_ = k•(RO – AO). During natural standing, healthy persons use a broad range of both R- and C-commands, reflected in the experimentally observed broad ranges of RO and k, and co-vary them to produce about the same M_ACT_ (Nardon et al. 2022).

Further, changes in these top-level {R; C} commands are distributed across abundant sets of {R; C} pairs at the level of effectors such as joints. Since most joints are spanned by multiple muscles, ultimately joint-level {R; C} pairs have to be distributed across control variables (thresholds of the stretch reflex, λs, Feldman 1966) for the individual muscles. Each of these few-to-many transformations can be viewed as abundant, i.e., affording numerous solutions that can be used to organized performance-stabilizing synergies (reviewed in Latash 2010, 2021). This sequence of neural commands is illustrated in Fig. 8B. Note that action at each level can be tentatively associated with specific groups of performance variables quantified in our study, such as COP shifts reflecting the whole-body {R; C} pair, M-modes reflecting the involvement of muscles crossing multiple joints, i.e., the combinations of {R; C} pairs at the joints, and EMGs reflecting the involvement of individual muscles.

During quiet standing, changes in the body joint configuration are relatively small (e.g., Hsu et al. 2007), and changes in muscle activation can be viewed as relatively adequate reflections of changes in the commands to the individual muscles (λ, Feldman 1966, 1986). Nonetheless, changes in the joint configuration space remain measurable and exhibit the characteristic synergic signature V_UCM_ > V_ORT_ (Hsu et al. 2007).

When a standing person prepares for a whole-body action, changes in the R- and C-commands at the M-mode level happen before visible changes in peripheral variables. These changes serve two purposes: To optimize the state of the body for the planned action (EPAs) and to attenuate stability of the COP coordinates in preparation to their upcoming changes (ASAs). Both have been observed in earlier studies (Klous et al. 2011; Falaki et al. 2023). In our study, the COP-stabilizing synergies were seen only for the COP-AP, and ASAs were under the level of statistical significance.

The absence of COP-ML synergies across all stepping conditions was not anticipated and requires discussion. Under predictable, self-paced stepping conditions on familiar surfaces, ML postural stability just before step initiation may represent a less dominant control goal than AP stability, which governs the primary biomechanical demands of weight transfer in the direction of stepping. It is possible that COP-ML synergies emerge preferentially under conditions that increase lateral instability, such as external perturbations, turns, narrow support surfaces, dual-task demands, or neurological impairment (Wang and Asaka 2008; Falaki et al. 2023).

Another possible explanation for why significant COP-ML synergies were not detected at steady state relates to the set of muscles included in the analysis. Most recorded muscles primarily contributed to sagittal-plane action (e.g., TA, SOL, RF, VL, BF, RA, ESL), whereas mediolateral balance regulation during gait initiation depends heavily on frontal-plane stabilizers, particularly hip abductors and adductors such as the gluteus medius. Since these muscles were not included, the elemental variable space may have underrepresented the neuromuscular components primarily responsible for COP-ML stabilization. Consequently, potential ML-specific synergy structures may not have been adequately captured by the present muscle set. Together, these findings suggest that steady-state synergic organization during step initiation primarily reflects the stabilization demands of pre-step steady-state posture in the AP direction and is not substantially shaped by the mechanical characteristics of the first step. The factors that may explain why significant ASAs were not detected are discussed in the following section.

### Interpreting anticipatory synergy adjustments (ASAs) findings

Anticipatory synergy adjustments (ASAs) were not detected in any condition or direction in the present study. Specifically, no significant drop in the synergy indices preceding step initiation was observed, regardless of surface height or step length. This finding contrasts with several previous studies reporting clear reductions in synergy indices prior to voluntary movements or perturbation-related actions, interpreted as transient destabilization of an existing synergy in preparation for rapid change in a performance variable (Olafsdottir et al. 2005; Shim et al. 2005; Klous et al. 2011; Wang et al. 2016, 2017).

Within the framework of the UCM hypothesis, ASAs have been described as feedforward adjustments characterized by a reduction in stability of a controlled variable approximately 200-400 ms before movement onset or predictable perturbation. These adjustments are thought to facilitate rapid modification of the performance variable by temporarily weakening the stabilizing synergy. Previous studies have demonstrated ASAs in a variety of contexts, including self-triggered load-release tasks, postural perturbations, quick body sway, multi-finger force production, and gaze-shift-related postural tasks (Kim et al. 2006; Klous et al. 2011; Wang et al. 2016, 2017; Falaki et al. 2023). Importantly, many of these tasks involved increased task complexity, temporal urgency, externally triggered perturbations, or explicit requirements for rapid COP reconfiguration.

For example, Klous and colleagues (2011) observed pronounced ASAs during self-triggered postural perturbation tasks involving rapid unloading and fast COP shifts. Similarly, Zhou and collaborators (2013) demonstrated that ASAs became larger and occurred earlier when participants could predict the timing of an upcoming force correction, suggesting that ASAs are particularly associated with conditions requiring rapid reorganization of motor coordination (Zhou et al. 2013). Studies involving neurological populations have also consistently shown impaired or reduced ASAs in conditions associated with deficient anticipatory control, particularly in Parkinson’s disease (Park et al. 2012b; Falaki et al. 2016; 2023; Freitas et al. 2020). In those studies, ASAs were interpreted as important neural mechanisms allowing controlled destabilization of an existing postural state before voluntary action.

In contrast, participants in the present study performed predictable, self-paced stepping tasks under relatively stable environmental conditions. Although surface height altered the magnitude and timing of EPAs, the stepping tasks did not require rapid or unexpected COP reconfiguration, nor did they involve external perturbations or strict temporal constraints. Under these circumstances, the CNS may have been able to initiate stepping while preserving the pre-existing synergic organization stabilizing COP-AP, suggesting that ASAs are not obligatory features of step initiation, but rather context-dependent mechanisms recruited when task demands require rapid modification of a stabilized variable. This interpretation is consistent with Piscitelli and colleagues (2017), who reported that the magnitude and timing of ASAs depend strongly on task characteristics and mechanical constraints, and that ASAs emerge more consistently under conditions involving elevated postural threat or the need for rapid transitions between stable states.

Taken together, at least three complementary, non-mutually exclusive factors may account for the absence of statistically significant ASAs across all conditions in the present study. First, the stepping task was predictable and self-paced, performed without an external perturbation or explicit temporal urgency; this contrasts with paradigms in which robust ASAs have been reported, which typically involve rapid, self-triggered or externally cued COP reconfiguration under time pressure. Second, the steady-state synergy stabilizing COP-AP was consistently present and did not differ across conditions, suggesting that this pre-existing stability may have been sufficient to support step initiation without requiring a transient destabilization of the synergy; under this view, ASAs may be recruited selectively when the pre-existing synergy would otherwise impede the required change in the performance variable, a condition that may not have been met in the present task. Third, the sample size (*n* = 16) and the small-to-moderate effect sizes observed for ASA magnitude across conditions (Cohen’s d ranging from approximately 0.03 to 0.51) indicate that the present design may have had limited statistical power to detect a genuine, smaller-magnitude anticipatory decline in the synergy index. As mentioned, these explanations are not mutually exclusive, and distinguishing among them will require future studies directly comparing self-paced and externally cued or time-constrained versions of the step-initiation task in larger samples.

It is also noteworthy that the preserved steady-state synergies observed here may have provided sufficient stability to support movement initiation without requiring synergy-level reorganization. Under predictable stepping conditions, the CNS appears to rely primarily on modulation of mechanical preparatory variables, COP trajectory and EPA timing, rather than on dismantling the existing stabilization structure, a pattern reflected in the strong EPA modulation by surface height observed despite the lack of statistically significant ASAs. Together, these results reinforce the idea that synergy-level destabilization and mechanical anticipatory adjustments represent partially independent control processes in motor preparation.

Collectively, the findings suggest that ASAs are not mandatory components of self-paced gait initiation in healthy young adults. Rather, their emergence appears to depend on the interplay among task complexity, postural threat, environmental instability, and the requirement for rapid postural reconfiguration. Under stable and predictable conditions, gait initiation can be successfully achieved through EPA modulation alone, while steady-state synergic organization remains largely unchanged.

### EPA modulation by stepping surface height

Surface height significantly influenced both the temporal and spatial characteristics of early postural adjustments (EPAs). Specifically, step-down initiation produced earlier EPA onset but lower COP displacements compared to step-up conditions, while level stepping produced intermediate responses. These findings indicate that descending movements required earlier preparation and greater anticipatory control before gait initiation.

From a biomechanical perspective, descending a step likely imposes greater postural threat, reduced mechanical safety margins, and increased demands for braking and control of the center of mass (COM). During step-down initiation, the CNS must prepare not only for forward progression, but also for controlled downward displacement of the COM under gravitational acceleration. This condition increases the challenge of maintaining dynamic stability during the transition from double support to single-limb support. Earlier and smaller EPAs may therefore reflect enhanced feedforward preparation necessary to safely unload the stepping limb and regulate COM motion prior to foot-off.

This interpretation is consistent with classical descriptions of gait initiation, in which preparatory COP shifts are viewed as mechanisms that create appropriate mechanical conditions for the upcoming movement. During gait initiation, COP displacement posteriorly and toward the stance limb generates the disequilibrium torque necessary for forward propulsion and lateral weight transfer (Crenna and Frigo 1991; Lepers and Brenière, 1995; Halliday et al. 1998). Krishnan and collaborators (2011) further proposed that EPAs represent a distinct preparatory stage occurring several hundred milliseconds prior to movement onset, functionally separate from the later anticipatory postural adjustments (APAs) traditionally observed approximately 100 ms before perturbation or movement initiation. According to this framework, EPAs primarily establish favorable mechanical conditions for the upcoming action, whereas APAs are more directly related to counteracting expected perturbations.

The stronger EPA responses observed during step-down, therefore, support the idea that EPAs are highly sensitive to the mechanical demands and stability constraints of the upcoming movement. Similar task-dependent scaling of preparatory postural adjustments has been reported in studies manipulating stepping speed, perturbation magnitude, or environmental constraints. Wang et al. demonstrated that faster and more demanding stepping tasks produced substantially larger anticipatory COP and EMG adjustments, despite relatively preserved synergy organization (Wang et al. 2006b). Likewise, studies of obstacle negotiation and stair descent have consistently shown increased preparatory control requirements relative to level walking, particularly due to the increased consequences of instability during downward transitions (Begg and Sparrow 2000; Chou et al. 2003; McFadyen and Winter 1988).

### Dissociation between synergic organization and COP trajectory control

One of the most important findings of the present study was the apparent dissociation between the modulation of EPAs and the relative stability of the synergy indices. Although surface height altered the timing and magnitude of early COP adjustments, steady-state multi-muscle synergies stabilizing COP-AP remained present and statistically unchanged across conditions, while significant ASAs were not detected. This dissociation suggests that stability-oriented synergic organization and trajectory-oriented anticipatory control are not obligatorily coupled: stepping surface height altered EPA characteristics but left COP-AP synergy strength statistically unchanged. This is a single, not a double, dissociation, and is therefore compatible with either separate neural mechanisms or a shared mechanism with different sensitivity across outputs. Identifying a complementary manipulation that affects synergy strength but not EPAs would be needed to distinguish between these possibilities.

In the present study, the synergy indices associated with pre-step steady-state posture appeared relatively robust and insensitive to task manipulations, whereas the mechanical expression of movement preparation was strongly modulated according to the expected biomechanical demands of the upcoming step. Similar dissociations between synergy structure and overt motor behavior have been previously described within the UCM framework.

Wang and colleagues (2006a) reported that multi-muscle synergies stabilizing COP remained comparatively stable despite substantial changes in the magnitude and rate of anticipatory postural adjustments during stepping tasks performed under different speed and reaction-time constraints (Wang et al. 2006a). Likewise, Krishnan and collaborators proposed that postural preparation involves partially independent components, including anticipatory synergy adjustments, changes in averaged muscle activation, and mechanical COP shifts (Krishnan, al al., 2011). Their findings demonstrated that changes in synergy indices may occur independently from mechanical adjustments, reinforcing the idea that stabilization of a performance variable and generation of its trajectory are not necessarily controlled through identical mechanisms.

Within this perspective, the preserved COP-AP synergies observed in the present study may primarily reflect mechanisms responsible for maintaining stability of quiet stance, whereas EPAs may represent a more flexible feedforward process directly involved in adapting gait initiation to environmental constraints. This interpretation is broadly consistent with theoretical views proposing hierarchical organization of motor control, in which higher-order stability constraints coexist with lower-level task-specific adaptations (Latash et al. 2007; Latash 2010). Within this theoretical framework, one possible interpretation is that steady-state synergies may ensure robustness of posture, while anticipatory adjustments may adapt the forthcoming movement to the expected mechanical context. However, the present findings cannot directly confirm this hierarchical neural organization and are equally consistent with alternative accounts.

Interestingly, step length did not significantly influence EPA characteristics. Although longer steps generally require greater propulsion and momentum generation, the manipulation of horizontal distance alone may not have substantially altered stability constraints during movement preparation. In contrast, changes in surface height directly modified gravitational and balance-related demands, especially during step-down transitions, thereby eliciting more pronounced preparatory adjustments, reflected as lower COP-AP displacement. This finding reinforces the idea that EPAs are particularly sensitive to perceived postural threat and dynamic stability requirements rather than simply to movement amplitude.

### Study limitations

Several limitations of the present study should be acknowledged. First, the sample size (*n* = 16) was relatively modest and was not based on an a priori power calculation, but rather on sample sizes used in previous studies from our laboratory and others employing similar single-step paradigms; this sample size might have limited statistical power to detect small but potentially meaningful effects, particularly for ASAs and COP-ML synergy indices, and replication with larger samples is warranted before definitive conclusions are drawn. Also, handedness and footedness were assessed using single-item self-report questions rather than a validated multi-item inventory.

Second, the recorded muscle set was representative of the sagittal-plane control and did not include major frontal-plane stabilizers, such as hip abductors and adductors (e.g., gluteus medius). This omission may have precluded the detection of COP-ML synergies. Relatedly, separate Jacobian matrices were estimated for the AP and ML directions, following established practice in UCM studies, but, since the participants controlled the COP within a bounded two-dimensional area rather than swaying in a single direction, these two directions may not be fully independent. A more comprehensive analysis would estimate a single Jacobian relating the two-dimensional COP vector to the four-dimensional M-mode space. Note, however, that such analysis would not be able to detect possible differences in the stabilization of COP shifts in the two directions. In addition, COP displacement, rather than center-of-mass (COM) kinematics, was used as the primary mechanical outcome; although COP and COM are mechanically related, they are not always redundant, and future studies incorporating a broader muscle set and full-body kinematic analysis would allow a more complete characterization of the postural strategies examined here.

Third, participants initiated the step from a forward-leaning posture achieved through an ankle-only strategy, used to ensure non-zero background muscle activity for the synergy analysis. This posture differs from an unconstrained upright stance. Forward lean angle was not directly measured, and the presence of non-zero muscle activity was verified qualitatively rather than against a quantitative threshold, precluding a more precise biomechanical characterization of this initial posture.

Fourth, although the three stepping-surface-height conditions were fully counterbalanced across participants, the step-length block was always performed after the height block, because the step-length targets depended on the step-level condition. Thus, a residual order effect cannot be fully excluded for the step-length comparisons. Also, only a single stepping height (17 cm) was tested for the step-up and step-down conditions and, although this height falls within the typical range of residential stair risers, findings may not generalize to other heights. Relatedly, participants also stood on a metal force platform while stepping onto wooden platforms, and differences in surface texture, compliance, and friction may have influenced sensory feedback independently of the height and length manipulations.

Fifth, the self-paced nature of the task, while not requiring a rapid reaction, may have introduced greater trial-to-trial timing variability than an externally cued paradigm, which could partly explain the lack of statistically significant ASAs. Whether ASAs would be present, reduced, or absent under a reaction-time version of this task remains an open question for future research, although the original study showed no ASAs in reaction-time tasks in contrast to clear ASAs in self-paced actions (Olafsdottir et al. 2005).

Finally, the study was conducted exclusively with healthy young adults, which limits the generalizability of the findings to other populations. Older adults, individuals with neurological conditions such as Parkinson’s disease, or those with a history of falls may exhibit fundamentally different synergic organization and anticipatory control strategies during step initiation, particularly under challenging surface conditions. Future studies should examine whether the dissociation between synergic organization and EPA modulation observed here is preserved or disrupted in these populations.

## Conclusions

The present study examined the influence of first-step constraints on multi-muscle synergies stabilizing the COP, anticipatory synergy adjustments, and early postural adjustments during step initiation in healthy young adults. The findings yield three main conclusions.

First, multi-muscle synergies stabilizing COP in the AP direction were consistently present during the steady-state phase preceding step initiation across all stepping conditions and both experimental blocks, and their strength was not influenced by the nature of the upcoming step. This finding supports the view that steady-state synergic organization reflects the demands of upright stance maintenance rather than anticipatory tuning to forthcoming step initiation constraints and is consistent with the Principle of Motor Abundance as a stable feature of postural control in healthy adults. Significant COP-ML synergies were not detected in any condition, suggesting that mediolateral postural stability during quiet stance may be achieved through either the participation of other muscles that were not assessed in this study (e.g., ankle invertors and evertors, hip adductors and abductors) or mechanisms other than multi-muscle co-variation, a question that warrants further investigation.

Second, significant ASAs were not detected in any condition or direction, suggesting that the neuromuscular system did not show a statistically detectable modulation of multi-muscle synergy strength in anticipation of step initiation regardless of the mechanical demands imposed by the first step. This finding contrasts with observations from upper limb voluntary movements and load-release tasks, where robust ASAs have been consistently reported, and raises the possibility that the postural-to-locomotor transition in step initiation may rely on EPA-based mechanisms rather than synergy-level anticipatory adjustments to manage the upcoming perturbation. Whether this reflects a fundamental difference between locomotor and non-locomotor voluntary movements, or a sensitivity limitation of the current paradigm, remains an open question.

Third, stepping surface height, but not step length, significantly influenced EPA onset timing and COP displacement magnitude in both the AP and ML directions. Specifically, the Step-Down condition was associated with earlier EPA onset and lower COP displacements compared with the Step-Up condition, while the Step-Level condition did not differ significantly from either. These findings indicate that descending steps impose a distinct postural challenge relative to ascending steps during step initiation, likely reflecting differences in the required direction and magnitude of COM displacement and the asymmetric biomechanical demands of downward versus upward stepping. The absence of significant step length effects on EPA outcomes suggests that, within the range tested, the postural system adapts to step length constraints without requiring measurable changes in the spatiotemporal organization of preparatory COP adjustments, at least in healthy young adults.

Taken together, these results advance our understanding of how first-step constraints shape the postural preparation for step initiation. The dissociation between UCM-based synergy measures and EPA outcomes across conditions suggests that these two aspects of postural control, synergic muscle organization and COP trajectory, may be functionally dissociable and differentially sensitive to task constraints. This behavioral dissociation does not, by itself, establish separate underlying neural control mechanisms.

## Data Availability

The datasets generated and/or analyzed during the current study are available from the corresponding author upon reasonable request.

## Abbreviations

AP: Anteroposterior
APA: Anticipatory postural adjustments
ASA: Anticipatory synergy adjustments
BF: Biceps femoris
C-command: Coactivation command
CNS: Central nervous system
COM: Center of mass
COP: Center of pressure
ΔCOP: Center of pressure displacements
ΔCOP_AP_: Center of pressure amplitude in the AP direction
ΔCOP_ML1_: COP-ML displacement from t_EPA_ to its maximum value in the direction of the leading leg
ΔCOP_ML2_: COP-ML displacement from its maximum value in the direction of the leading leg to t_0_ (Moment of right foot lift-off)
DOF: Degrees of freedom
EMG: Surface electromyographic activity
EMG-pk: Peak EMG
EPA: Early postural adjustment
ESL: Lumbar erector spinae
J: Jacobian matrix
M-modes: Muscle modes
ML: Mediolateral
ORT: Orthogonal
PCA: Principal component analysis
Pref-Step: Preferred step length
p-_FDR_: FDR-adjusted *p*-values are reported as *p*-_FDR_
QS: Quiet standing
R-command: Reciprocal command
RA: Rectus abdominis
RF: Rectus femoris
RM-ANOVA: Repeated-measures analysis of variance
RMS: Root mean square
SOL: Soleus
SS: Steady-state
TA: Tibialis anterior
t_0_: Moment of right foot lift-off
t_EPA_: Onset of early postural adjustment
TFL: Tensor fasciae latae
UCM: Uncontrolled manifold
VL: Vastus lateralis
V_ORT_: Variance orthogonal to the UCM
VS: Voluntary sway
V_TOT_: Total variance per dimension
V_UCM_: Variance within the UCM
ΔM: Muscle mode magnitudes
ΔV: Synergy index
ΔV_Z_: Fisher z-transformed synergy index
ΔΔV_Z_: Difference between ΔVZ at SS and ΔVZ at t0
η²p: Partial eta squared

## References

[CR67] Begg RK, Sparrow WA (2000) Gait characteristics of young and older individuals negotiating a raised surface: implications for the prevention of falls. J Gerontol A Biol Sci Med Sci 55(3), M147-M154. 10.1093/gerona/55.3.M147 10795727

[CR71] Bernstein NA (1947) On the construction of movements. Moscow: Medgiz (in Russian). English translation is in: Latash ML (Ed.) (2020) Bernstein’s construction of movements. Routledge: Abingdon, UK.

[CR1] Bernstein N (1967) The co-ordination and regulation of movements. Pergamon

[CR61] Belenkiy VY, Gurfinkel VS, Paltsev YI (1967) Elements of control of voluntary movements. Biofizika. 10:135–141. 5623488

[CR62] Bouisset S, Zattara M(1987) Biomechanical study of the programming of anticipatory postural adjustments associated with voluntary movement. J Biomech. 20:735–742. 10.1016/0021-9290(87)90052-2 3654672

[CR2] Brenière Y, Do MC (1991) Control of gait initiation. J Mot Behav 23(4):235–240. 10.1080/00222895.1991.9942034 14766505

[CR3] Brenière Y, Do MC, Bouisset S (1987) Are dynamic phenomena prior to stepping essential to walking? J Mot Behav 19(1):62–76. 10.1080/00222895.1987.10735400 23944913

[CR4] Chou LS, Kaufman KR, Hahn ME, Brey RH (2003) Medio-lateral motion of the center of mass during obstacle crossing distinguishes elderly individuals with imbalance. Gait Posture 18(3):125–133. 10.1016/s0966-6362(02)00067-x 14667945

[CR5] Cohen J (1988) Statistical power analysis for the behavioral sciences, 2nd edn. Lawrence Erlbaum Associates

[CR6] Corcos DM, Gottlieb GL, Latash ML, Almeida GL, Agarwal GC (1992) Electromechanical delay: an experimental artifact. J Electromyogr Kinesiol 2(2):59–68. 10.1016/1050-6411(92)90017-D 20719599

[CR7] Crenna P, Frigo C (1991) A motor programme for the initiation of forward-oriented movements in humans. J Physiol 437:635–653. 10.1113/jphysiol.1991.sp018616 PMC1180068 1890653

[CR63] Criswell E (2010) Cram’s Introduction to Surface Electromyography. Second edition. Jones & Bartlett Learning.

[CR8] Danna-dos-Santos A, Slomka K, Zatsiorsky VM, Latash ML (2007) Muscle modes and synergies during voluntary body sway. Exp Brain Res 179(4):533–550. 10.1007/s00221-006-0812-0 17221222

[CR9] de Freitas PB, Freitas SMSF, Prado-Rico JM, Lewis MM, Du G, Yanosky JD, Latash ML (2022) Synergic control in asymptomatic welders during multi-finger force exertion and load releasing while standing. Neurotoxicology 93:324–336. 10.1016/j.neuro.2022.10.012 PMC10398836 36309163

[CR66] Falaki A, Huang X, Lewis MM, Latash ML (2016) Impaired synergic control of posture in Parkinson’s patients without postural instability. Gait Posture 44:209–215. 10.1016/j.gaitpost.2015.12.035 PMC4806225 27004660

[CR11] Falaki A, Huang X, Lewis MM, Latash ML (2017) Dopaminergic modulation of multi-muscle synergies in postural tasks performed by patients with Parkinson’s disease. J Electromyogr Kinesiol 33:20–26. 10.1016/j.jelekin.2017.01.002 PMC5357450 28110044

[CR10] Falaki A, Cuadra C, Lewis MM, Prado-Rico JM, Huang X, Latash ML (2023) Multi-muscle synergies in preparation for gait initiation in Parkinson’s disease. Clin Neurophysiol 154:12–24. 10.1016/j.clinph.2023.06.022 37524005

[CR12] Feldman AG (1966) Functional tuning of the nervous system with control of movement or maintenance of a steady posture. II. Controllable parameters of the muscle. Biophysics 11:565–578

[CR13] Feldman AG (1986) Once more on the equilibrium-point hypothesis (λ-model) for motor control. J Mot Behav 18:17–54. 10.1080/00222895.1986.10735369 15136283

[CR14] Feldman AG (2015) Referent control of action and perception: challenging conventional theories in behavioral science. Springer, NY. 10.1007/978-1-4939-2736-4

[CR15] Freitas SMSF, Duarte M (2012) Joint coordination in young and older adults during quiet stance: effect of visual feedback of the center of pressure. Gait Posture 35(1):83–87. 10.1016/j.gaitpost.2011.08.011 21962847

[CR16] Freitas SMSF, de Freitas PB, Falaki A, Corson T, Lewis MM, Huang X, Latash ML (2020) Synergic control of action in levodopa-naïve Parkinson’s disease patients: II. Multi-muscle synergies stabilizing vertical posture. Exp Brain Res 238(12):2931–2945. 10.1007/s00221-020-05947-z PMC7644647 33068173

[CR17] Gélat T, Brenière Y (2000) Adaptation of the gait initiation process for stepping on to a new level using a single step. Exp Brain Res 133(4):538–546. 10.1007/s002210000452 10985688

[CR18] Gélat T, Le Pellec A (2007) Why anticipatory postural adjustments in gait initiation need to be modified when stepping up onto a new level? Neurosci Lett 429(1):17–21. 10.1016/j.neulet.2007.09.054 17964073

[CR19] Halliday SE, Winter DA, Frank JS, Patla AE, Prince F (1998) The initiation of gait in young, elderly, and Parkinson’s disease subjects. Gait Posture 8(1):8–14. 10.1016/S0966-6362(98)00020-4 10200394

[CR20] Hsu WL, Scholz JP, Schöner G, Jeka JJ, Kiemel T (2007) Control and estimation of posture during quiet stance depends on multijoint coordination. J Neurophysiol 97(4):3024–3035. 10.1152/jn.01142.2006 17314243

[CR69] Kaiser HF (1960) The application of electronic computers to factor analysis. Educ Psychol Measur 20(1):11.

[CR21] Kim SW, Shim JK, Zatsiorsky VM, Latash ML (2006) Anticipatory adjustments of multi-finger synergies in preparation for self-triggered perturbations. Exp Brain Res 174(4):604–612. 10.1007/s00221-006-0505-8 16724179

[CR64] Klous M, Danna-dos-Santos A, Latash ML (2010) Multi-muscle synergies in a dual postural task: Evidence for the principle of superposition. Exp Brain Res 202:457–471 10.1007/s00221-009-2153-2 PMC2852137 20047089

[CR22] Klous M, Mikulic P, Latash ML (2011) Two aspects of feedforward postural control: anticipatory postural adjustments and anticipatory synergy adjustments. J Neurophysiol 105(5):2275–2288. 10.1152/jn.00665.2010 PMC3094180 21389305

[CR23] Krishnamoorthy V, Goodman S, Zatsiorsky V, Latash ML (2003a) Muscle synergies during shifts of the center of pressure by standing persons: identification of muscle modes. Biol Cybern 89(2):152–161. 10.1007/s00422-003-0419-5 12905043

[CR24] Krishnamoorthy V, Latash ML, Scholz JP, Zatsiorsky VM (2003b) Muscle synergies during shifts of the center of pressure by standing persons. Exp Brain Res 152(3):281–292. 10.1007/s00221-003-1574-6 12904934

[CR25] Krishnamoorthy V, Latash ML, Scholz JP, Zatsiorsky VM (2004) Muscle modes during shifts of the center of pressure by standing persons: effect of instability and additional support. Exp Brain Res 157(1):18–31. 10.1007/s00221-003-1812-y 14985897

[CR26] Krishnan V, Aruin AS, Latash ML (2011) Two stages and three components of the postural preparation to action. Exp Brain Res 212:47–63. 10.1007/s00221-011-2694-z PMC3732002 21537967

[CR27] Krishnan V, Latash ML, Aruin AS (2012) Early and late components of feed-forward postural adjustments to predictable perturbations. Clin Neurophysiol 123(5):1016–1026. 10.1016/j.clinph.2011.09.014 PMC3258385 21983281

[CR28] Latash ML (2010) Motor synergies and the equilibrium-point hypothesis. Motor Control 14:294–322. 10.1123/mcj.14.3.294 PMC2921643 20702893

[CR29] Latash ML (2012) The bliss (not the problem) of motor abundance (not redundancy). Exp Brain Res 217(1):1–5. 10.1007/s00221-012-3000-4 PMC3532046 22246105

[CR30] Latash ML (2016) Towards physics of neural processes and behavior. Neurosci Biobehav Rev 69:136–146. 10.1016/j.neubiorev.2016.08.005 PMC5030181 27497717

[CR31] Latash ML (2021) One more time about motor (and non-motor) synergies. Exp Brain Res 239(10):2951–2967. 10.1007/s00221-021-06188-4 34383080

[CR32] Latash ML, Huang X (2015) Neural control of movement stability: lessons from studies of neurological patients. Neuroscience 301:39–48. 10.1016/j.neuroscience.2015.05.075 PMC4504804 26047732

[CR34] Latash ML, Zatsiorsky VM (1993) Joint stiffness: myth or reality? Hum Mov Sci 12(6):653–692. 10.1016/0167-9457(93)90010-M

[CR33] Latash ML, Scholz JP, Schöner G (2007) Toward a new theory of motor synergies. Motor Control 11(3):276–308. 10.1123/mcj.11.3.276 17715460

[CR35] Lepers R, Breniere Y (1995) The role of anticipatory postural adjustments and gravity in gait initiation. Exp Brain Res 107(1):118–124. 10.1007/bf00228023 8751069

[CR36] Massion J (1984) Postural changes accompanying voluntary movements: normal and pathological aspects. Hum Neurobiol 2(4):261–267 6715210

[CR70] Massion J (1992) Movement, posture and equilibrium: Interaction and coordination. Prog Neurobiol 38:35–56. 10.1016/0301-0082(92)90034-c 1736324

[CR38] McFadyen BJ, Winter DA (1988) An integrated biomechanical analysis of normal stair ascent and descent. J Biomech 21(9):733–744. 10.1016/0021-9290(88)90282-5 3182877

[CR37] McIlroy WE, Maki BE (1999) The control of lateral stability during rapid stepping reactions evoked by antero-posterior perturbation: does anticipatory control play a role? Gait Posture 9(3):190–198. 10.1016/S0966-6362(99)00013-2 10575080

[CR39] Melzer I, Kurz I, Shahar D, Levi M, Oddsson LIE (2007) Application of the voluntary step execution test to identify elderly fallers. Age Ageing 36(5):532–537. 10.1093/ageing/afm068 17545207

[CR40] Nadeau S, McFadyen BJ, Malouin F (2003) Frontal and sagittal plane analyses of the stair climbing task in healthy adults aged over 40 years: what are the challenges compared to level walking? Clin Biomech Elsevier Ltd 18(10):950–959. 10.1016/s0268-0033(03)00179-7 14580839

[CR41] Nardon M, Pascucci F, Cesari P, Bertucco M, Latash ML (2022) Synergies stabilizing vertical posture in spaces of control variables. Neuroscience 500:79–94. 10.1016/j.neuroscience.2022.08.006 35952997

[CR42] Olafsdottir H, Yoshida N, Zatsiorsky VM, Latash ML (2005) Anticipatory covariation of finger forces during self-paced and reaction time force production. Neurosci Lett 381(1–2):92–96. 10.1016/j.neulet.2005.02.003 PMC2827154 15882796

[CR44] Park J, Singh T, Zatsiorsky VM, Latash ML (2012a) Optimality vs. variability: effect of fatigue in multi-finger redundant tasks. Exp Brain Res 216(4):591–607. 10.1007/s00221-011-2963-x PMC3623544 22130781

[CR45] Park J, Wu YH, Lewis MM, Huang X, Latash ML (2012b) Changes in multifinger interaction and coordination in Parkinson’s disease. J Neurophysiol 108(3):915–924. 10.1152/jn.00043.2012 PMC3424084 22552184

[CR43] Park E, Reimann H, Schöner G (2016) Coordination of muscle torques stabilizes upright standing posture: an UCM analysis. Exp Brain Res 234(6):1757–1767. 10.1007/s00221-016-4576-x 26879770

[CR46] Piscitelli D, Falaki A, Solnik S, Latash ML (2017) Anticipatory postural adjustments and anticipatory synergy adjustments: preparing to a postural perturbation with predictable and unpredictable direction. Exp Brain Res 235(3):713–730. 10.1007/s00221-016-4835-x PMC5316309 27866261

[CR47] Prilutsky BI, Zatsiorsky VM (2002) Optimization-based models of muscle coordination. Exerc Sport Sci Rev 30(1):32–38. 10.1097/00003677-200201000-00007 PMC2821033 11800497

[CR48] Santos-Dias M, Russo-Junior DV, de Freitas PB (2026) Multi-joint synergies stabilize anteroposterior and vertical toe clearance during single-step ascent. J Biomech. 10.1016/j.jbiomech.2026.113461 42447767

[CR49] Scholz JP, Schöner G (1999) The uncontrolled manifold concept: identifying control variables for a functional task. Exp Brain Res 126(3):289–306. 10.1007/s002210050738 10382616

[CR50] Scholz JP, Schöner G (2014) Use of the uncontrolled manifold (UCM) approach to understand motor variability, motor equivalence, and self-motion. In: Levin MF (ed) Progress in motor control. Springer, pp. 91–100. 10.1007/978-1-4939-1338-1_7 25330887

[CR51] Scholz JP, Park E, Jeka JJ, Schöner G, Kiemel T (2012) How visual information links to multijoint coordination during quiet standing. Exp Brain Res 222(3):229–239. 10.1007/s00221-012-3210-9 22918606

[CR65] Shim JK, Olafsdottir H, Zatsiorsky VM, Latash ML (2005) The emergence and disappearance of multidigit synergies during force production tasks. Exp Brain Res 164:260–270. 10.1007/s00221-005-2248-3 PMC2826980 15770477

[CR52] Wang Y, Asaka T (2008) Muscle synergies involved in shifts of the center of pressure while standing on a narrow support. Brain Res Bull 76(1–2):16–25. 10.1016/j.brainresbull.2007.12.010 18395606

[CR56] Wang Y, Zatsiorsky VM, Latash ML (2005) Muscle synergies involved in shifting the center of pressure while making a first step. Exp Brain Res 167(2):196–210. 10.1007/s00221-005-0012-3 16034579

[CR57] Wang Y, Zatsiorsky VM, Latash ML (2006a) Muscle synergies involved in preparation to a step made under the self-paced and reaction time instructions. Clin Neurophysiol 117(1):41–56. 10.1016/j.clinph.2005.09.002 16364687

[CR53] Wang Y, Asaka T, Zatsiorsky VM, Latash ML (2006b) Muscle synergies during voluntary body sway: combining across-trials and within-a-trial analyses. Exp Brain Res 174(4):679–693. 10.1007/s00221-006-0513-8 16710681

[CR54] Wang Y, Watanabe K, Asaka T (2016) Muscle synergies underlying control of taking a step during support surface translation. Eur J Appl Physiol 116(2):301–309. 10.1007/s00421-015-3282-x 26480851

[CR55] Wang Y, Watanabe K, Asaka T (2017) Aging effect on muscle synergies in stepping forth during a forward perturbation. Eur J Appl Physiol 117(1):201–211. 10.1007/s00421-016-3514-8 28004203

[CR58] Winter DA, Prince F, Frank JS, Powell C, Zabjek KF (1996) Unified theory regarding A/P and M/L balance in quiet stance. J Neurophysiol 75(6):2334–2343. 10.1152/jn.1996.75.6.2334 8793746

[CR59] Yamada H, Shinya M (2021) Variability in the center of mass state during initiation of accurate forward step aimed at targets of different sizes. Front Sports Act Living 3:691307. 10.3389/fspor.2021.691307 PMC8416920 34490423

[CR60] Zhou T, Wu YH, Bartsch A, Cuadra C, Zatsiorsky VM, Latash ML (2013) Anticipatory synergy adjustments: preparing a quick action in an unknown direction. Exp Brain Res 226(4):565–573. 10.1007/s00221-013-3469-5 PMC3634869 23494385

